# Global birth prevalence and mortality from inborn errors of metabolism: a systematic analysis of the evidence

**DOI:** 10.7189/jogh.08.021102

**Published:** 2018-12

**Authors:** Donald Waters, Davies Adeloye, Daisy Woolham, Elizabeth Wastnedge, Smruti Patel, Igor Rudan

**Affiliations:** Centre for Global Health Research, The Usher Institute for Population Health Sciences and Informatics, University of Edinburgh, Edinburgh Scotland, UK; * These authors contributed equally.

## Abstract

**Background:**

Inborn errors of metabolism (IEM) are a group of over 500 heterogeneous disorders resulting from a defect in functioning of an intermediate metabolic pathway. Individually rare, their cumulative incidence is thought to be high, but it has not yet been estimated globally. Although outcomes can often be good if recognised early, IEM carry a high fatality rate if not diagnosed. As a result, IEM may contribute significantly to the burden of non-communicable childhood morbidity.

**Methods:**

We conducted a systematic literature review of birth prevalence and case fatality of IEM globally, with search dates set from 1980 to 2017. Using random-effects meta-analysis, we estimated birth prevalence of separate classes of IEM and all-cause IEM, split by geographical region. We also estimated levels of parental consanguinity in IEM cases and global case fatality rates and resultant child deaths from all-cause IEM.

**Findings:**

49 studies met our selection criteria. We estimate the global birth prevalence of all-cause IEM to be 50.9 per 100 000 live births (95% confidence intervals (CI) = 43.4-58.4). Regional pooled birth prevalence rates showed the highest rates of IEM to be in the Eastern Mediterranean region (75.7 per 100 000 live births, 95% CI = 50.0-101.4), correlating with a higher observed rate of parental consanguinity in studies from this area. We estimate case fatality rates to be 33% or higher in low- and middle-income countries (LMICs), resulting in a minimum of 23 529 deaths from IEM per year globally (95% CI = 20 382-27 427), accounting for 0.4% of all child deaths worldwide.

**Conclusions:**

IEM represent a significant cause of global child morbidity and mortality, comprising a notable proportion of child deaths currently not delineated in global modelling efforts. Our data highlight the need for policy focus on enhanced laboratory capacity for screening and diagnosis, community interventions to tackle parental consanguinity, and increased awareness and knowledge regarding management of IEM, particularly in LMICs.

As outcomes improve in many areas of the world for common infectious causes of child morbidity and mortality, the contribution of other less common illnesses to the burden of disease in those under 5 years old is receiving increased attention [1,2]. One group of such diseases are the ‘inborn errors of metabolism’ (IEM), a heterogeneous group of over 500 disorders, individually rare but with a likely high cumulative incidence and potential to result in substantial mortality or long-term morbidity [3].

IEM are defined as monogenic diseases resulting in deficient activity of an individual enzyme, structural protein or transporter molecule in an intermediate metabolic pathway [4,5]. This deficiency can present clinically in a wide variety of ways, ranging from non-specific chronic issues such as childhood delay in attaining development milestones, to acute decompensation in the neonatal period with severe treatment-refractory metabolic acidosis or hypoglycaemia [2]. Most disorders are treatable if diagnosed early but can be rapidly fatal without prompt recognition [6]. Resulting from the heterogeneity in clinical presentation, which can overlap with many other diseases, diagnosis of IEM can prove difficult, and is often missed. Some IEM such as phenylketonuria (PKU) have been part of newborn screening tests in many countries for over 30 years, however rarer conditions are often not part of routine screening [4,6]. Diagnosis of these conditions often requires a high index of clinical suspicion combined with relatively novel sophisticated diagnostic investigations such as tandem mass spectrometry [7,8]. Diagnostic difficulty is more marked in low- and middle-income countries (LMICs) where access to advanced diagnostic approaches is limited due to resource constraints [9]. This is particularly of concern in areas with high levels of parental consanguinity, a known risk-factor for monogenic disorders such as IEM [7].

As of yet, there are no global estimates of the burden of morbidity or mortality associated with IEM, with IEM remaining part of the ‘other’ diseases section of estimates from the Interagency Group on Child Mortality Estimation (IGME), Maternal and Child Epidemiology Estimation (MCEE) or Global Burden of Disease (GBD) studies, and other similar large-scale global modelling efforts [1,10]. We are of the opinion that IEM likely contribute a significant proportion of this currently poorly understood subdivision of disease burden. For example, even based on an assumption that IEM incidence is as low as 1 in 10 000 births globally, hypothetically, out of 140 million live births per year globally, 14 000 children will be born with IEM. Given known high case fatality rates, particularly in LMICs due to limitations in diagnosis and management, IEM would result in a large number of under 5 deaths each year [4,5]. Without greater understanding of the contribution of IEM to the global disease burden, both as a whole and individually, the potential for advocacy of IEM as an important concern for policy makers and development of targeted screening and treatment approaches will remain severely limited. In an effort to combat this, we aimed in this study to provide global estimates of birth prevalence of IEM, and resultant mortality, in children under 5 years of age.

## METHODS

### Search strategy

A systematic search of published literature was performed on databases Medline, EMBASE and Global Health for the years 1980-2017 using the search terms shown in Appendix 1 in **Online Supplementary Document(Online Supplementary Document).** This was conducted in parallel by two separate reviewers to minimise risk of bias. Studies were evaluated using the selection criteria shown in **Table 1** and case definition criteria.

**Table 1:** Selection criteria for literature review

Inclusion criteria:
Studies published 1980-2017 globally that referred to inborn errors of metabolism (IEM) among children
Studies that directly attempted to estimate birth prevalence and/or under-5 mortality rate of all or specific classes of IEM
Studies that provided information on determinants of IEM aetiology/occurrence, consanguinity rates, clinical features, management, case fatality rates and/or outcomes
**Exclusion criteria:**
Studies that did not estimate the birth prevalence and/or under-five mortality of IEM
Studies that were review articles, viewpoints and commentaries
Studies that did not report total live births or relevant denominator from which birth prevalence and/or under-five mortality of IEM could be estimated
Studies with ambiguous study design or analysis
Studies without active follow-up periods
Studies with unclear defined or inconsistently applied case definitions.

### IEM case definitions

Diagnoses of IEM have been based on varying newborn screening tests, including tandem mass spectrometry, gas chromatography, traditional enzyme assays, radiological investigations and/or clinical symptoms. To ensure relatively uniform approach to case definitions in this review, we checked that studies reported cases and deaths from all causes of IEM based on the International Classification of Diseases 10 ((ICD-10), ie, E70-E90) and Online Mendelian Inheritance in Man (OMIM). Eight IEM classes were identified from studies. Other classes of IEM returned too few data points and were not included in the meta-analysis (**Table 2**).

**Table 2:** Classes of inborn errors of metabolism (IEM) identified from studies

IEM class	Examples
Amino acid disorders	Phenylketonuria, homocystinuria
Organic acid disorders	Propionic aciduria, methyl malonic aciduria, isovaleric aciduria, biotinidase deficiency
Fatty acid disorders	Short or medium chain acyl-coenzyme A dehydrogenase deficiency (SCAD, MCAD)
Lysosomal storage disorders	Sphyngolipidoses (Fabry, Farber, Gauher and Niemann-Pick diseases), mucolipidoses, oligosaccharidoses (fucosidosis, mannosidosis)
Carbohydrate metabolism disorders	Galactosemia, Pompe’s disease (glycogen);
Urea cycle disorders	Citrullinemia, argininemia
Mitochondrial disorders	Leigh syndrome
Peroxisomal disorders	Zellweger syndrome, Refsum syndrome
Others*	Purine and pyrimidine disorders, metal disorders, porphyria and haematological disorders, lipid disorders and myelin metabolism disorders

*Returned too few data points to be included in the meta-analysis.

### Data extraction

To minimise risk of bias, a parallel search (and double extraction) was conducted by two independent reviewers (DW and DA). Data were abstracted systematically on period of study, location, WHO region, study population, mean age or age range, number of IEM cases, birth prevalence rates, number of deaths, and case fatality rates (CFR) from IEM. For studies conducted on the same study site, population or cohort, the first chronologically published study was selected, and all additional data from additional studies were added in subsequently.

### Quality assessment

Studies were assessed for quality using a modified Grading of Recommendations Assessment, Development and Evaluation (GRADE) framework [11]. Quality of studies was assessed under four criteria: i) study design – assesses adherence to standardized screening for determining IEM, and validating data before data entry; ii) sampling strategy – assesses if the sampling was representative of a larger population in the region of study; iii) statistical analysis – assesses the appropriateness of statistical and analytical methods in determining the outcome measure/s; and iv) study limitations – assesses explicit description of the study limitation with reference to each of the first three criteria (study design, sampling and analysis). Studies that were graded as high, moderate, low and very low quality, respectively (see Appendix 2 in **Online Supplementary Document(Online Supplementary Document)** for details of grading). All ‘*very low’* quality studies were excluded as were the majority of ‘*low’* quality studies, some being included in quantitative analysis on the basis of well represented study designs.

### Data analysis

A random effects meta-analysis (DerSimonian and Laird method) [12] was conducted on extracted crude birth prevalence rates for all and specific classes of IEM, and on reported numbers of IEM deaths and CFR. Standard errors were determined from the reported crude estimates and live births (or other relevant denominators when appropriate), assuming a binominal (or Poisson) distribution. Heterogeneity between studies was assessed using I-squared (I^2^) statistics. Meta-estimates for birth prevalence rates were also split by WHO region. Birth prevalence and CFR meta-estimates were combined with United Nations Population Division (UNPD) data for the period 2015-2020 to estimate childhood deaths from IEM.

## RESULTS

**Figure 1:**
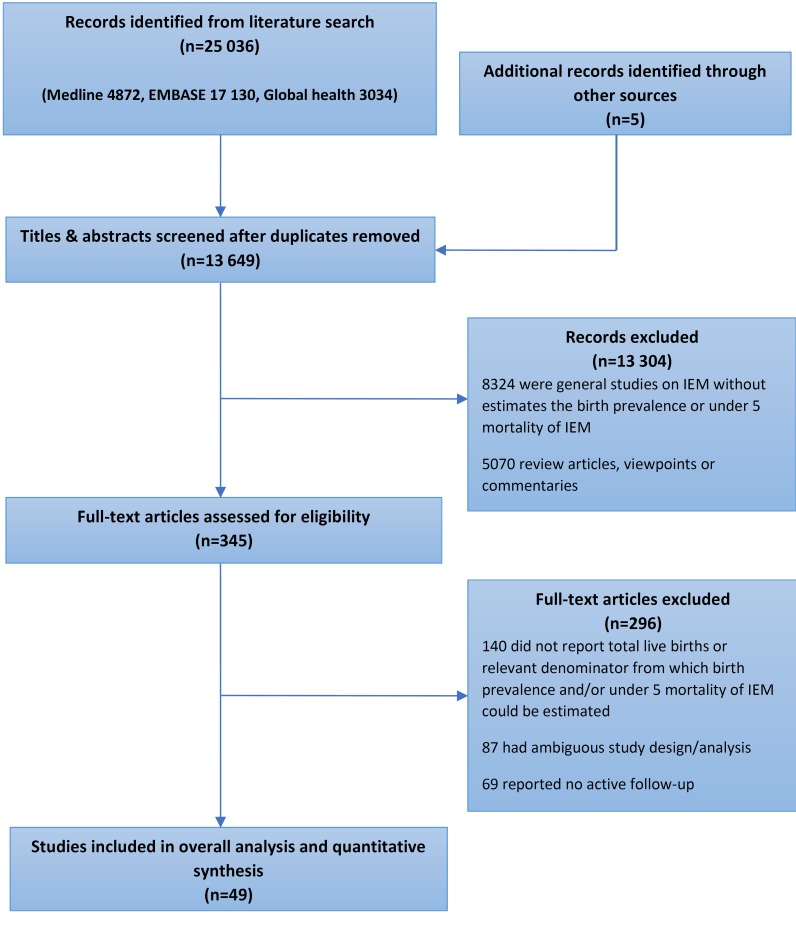
PRISMA flowchart of literature search.

Our searches returned 25036 records. Following exclusion of duplicates and application of selection criteria, 49 studies were included in the final analysis. Search results are shown in **Figure 1**.

### Study characteristics

Of the 49 studies included in the analysis, 33 reported estimates for all classes of IEM, with the remaining 16 studies only reporting for specific classes of IEM. The selected studies were from 38 countries globally. From the 33 studies that provided estimates for all classes of IEM, study period ranged from 2000 to 2014, with over 20 studies conducted after 2010. The majority of studies (n = 13) were conducted in the WHO Western Pacific region (*WPR A*-5, *WPR B*-8). There were seven studies in each of European and Eastern Mediterranean regions, while the American and the South East Asian regions had four and two studies, respectively. There were no studies from sub-Saharan Africa. **Table 3** shows study characteristics for studies reporting estimates for all classes of IEM, study characteristics of condition-specific studies are shown in Appendix 3 and 4 in **Online Supplementary Document(Online Supplementary Document).**

**Table 3:** Characteristics of studies reporting estimates for all classes of inborn errors of metabolism (IEM)*

Author	Location	WHO Region	Setting	Period of study	Consanguinity rate (%)	Total live births	IEM cases in cohort	Birth Prevalence /10^5^ LB	Deaths in birth cohort†
AlObaidy [13]	Libya, Tripoli	EMR B	Urban	Jan 2001 – Dec 2012	86	156 006	107	68.59	35
Al Bu Ali et al [14]	Saudi Arabia, Al Ahsa	EMR B	Urban-Rural	Apr 2006 – Apr 2009	34	38 001	48	126.31	
Goel et al [15]	Australia, Victoria	WPR A	Urban	Jan 1997 – Jan 2007					90
Huang et al [16]	China, Zhejiang Province	WPR B	Urban-Rural	Jan 2008 – Jan 2011	2		62		5
Al Riyami et al [17]	Oman, Al Khoudh	EMR B	Urban-Rural	May 1998 – Jul 2008	81	241 757	119	49.22	
Feuchtbaum et al [18]	USA, California	AMR A	Urban	Jul 2005 – Jul 2010		2 282 138	698	30.60	
Golbahar et al [19]	Bahrain	EMR B	Urban-Rural	Jan 2008 – Dec 2011	84	66 565	25	37.56	
Kamate et al [20]	India, Karnataka	SEAR D	Urban-Rural	Aug 2007 – Sep 2008	73		11		4
Moammar et al [21]	Saudi Arabia, Dhahran Eastern Province	EMR B	Urban-Rural	Jan 1983 – Jan 2008	93	165 530	248	149.82	
Applegarth et al [22]	Canada, British Columbia	AMR A	Urban	Jan 1969 – Jan 1996	6	1 142 912	249	40.00	
Dionisi-Vici et al [23]	Italy, national survey	EUR A	Urban-Rural	Jan 1985 – Dec 1997	3	5 336 730	1935	36.26	356
Sanderson et al [5]	UK, West Midlands	EUR A	Urban	Jan 1999 – Dec 2003	11	310 510	396	127.55	
Ibarra- Gonzalez et al [24]	Mexico, Mexico City	AMR B	Urban	Jan 2007 – Dec 2012	18		11		7
Nagaraja et al [25]	India, Southern states	SEAR D	Urban-Rural	Jan 2007 – Dec 2009	62		113		
Han et al [26]	China, Shanghai	WPR B	Urban	Jan 2002 – Oct 2006			212		26
Huang et al [27]	China, Zhejiang province	WPR B	Urban-Rural	Jan 2008 – Jan 2011		129 415	23	17.77	
Shigematsu et al [28]	Japan, Fukui	WPR A	Urban-Rural	Apr 1997 – Jul 2001		102 200	12	11.74	
Tu et al [29]	China, Beijing	WPR B	Urban	Jan – Dec 2009		8211	8	97.43	4
Yoon et al [30]	South Korea, Seoul	WPR B	Urban	Apr 2001 – Mar 2004		79 179	28	35.70	
Hadj-Taieb et al [31]	Tunisia, Tunis	EMR B	Urban	Jan 1987 – Dec 2009	82	946 404	370	39.10	
Klose et al [32]	Germany	EUR A	Urban-Rural	Jan 1999- Dec 2000		844 575	57	6.75	8
Noraihan et al [33]	Malaysia, Kuala Lumpur	WPR B	Urban	Jan 1990 – June 2000		34 109	4	11.73	
Tu, Wen-Jun [34]	China, Mainland	WPR B	Urban-Rural	Jan 2000 – Dec 2009		400 000	652	163.00	
Couce et al [35]	Spain, Galicia	EUR A	Urban	Jul 2000 – Jul 2010		210 165	137	48.54	4
Frazier et al [36]	USA, North Carolina	AMR A	Urban	Jul 1997 – Jul 2005		944 078	219	23.26	7
Kasper et al [37]	Austria, National	EUR A	Urban-Rural	Apr 2002 – Dec 2009		622 489	218	35.03	
Niu et al [38]	Taiwan	WPR B	Urban-Rural	Mar 2000 – Jun 2009		1495 132	170	16.08	
Schulze et al [8]	Germany	EUR A	Urban-Rural	Apr 1998 – Sep 2001		250 000	106	42.40	3
Selim et al [39]	Egypt	EMR D	Urban-Rural	Jun 2008 – Jun 2013	88	1 100 000	203	18.45	
Tan et al [40]	Singapore	WPR A	Urban	Jan 1992 –Jan 2005		40 800	127	311.27	
Vilarinho et al [41]	Portugal, national	EUR A	Urban-Rural	Jan 2005 – Jan 2009		316 243	132	41.74	
Wilcken et al [6]	Australia, New South Wales / Sydney	WPR A	Urban	Apr 1998 – Mar 2002		362 000	225	62.15	
Wilcken et al [42]	Australia, national	WPR A	Urban-Rural	Jan 1994 – Jan 2002		1 551 200	115	7.41	26

*See **Online Supplementary Document(Online Supplementary Document)**, Appendix 3 and 4 for details of all studies.

†Population denominator not provided in these birth cohorts, hence not included in meta-analysis

### Birth prevalence of IEM

**Figure 2:**
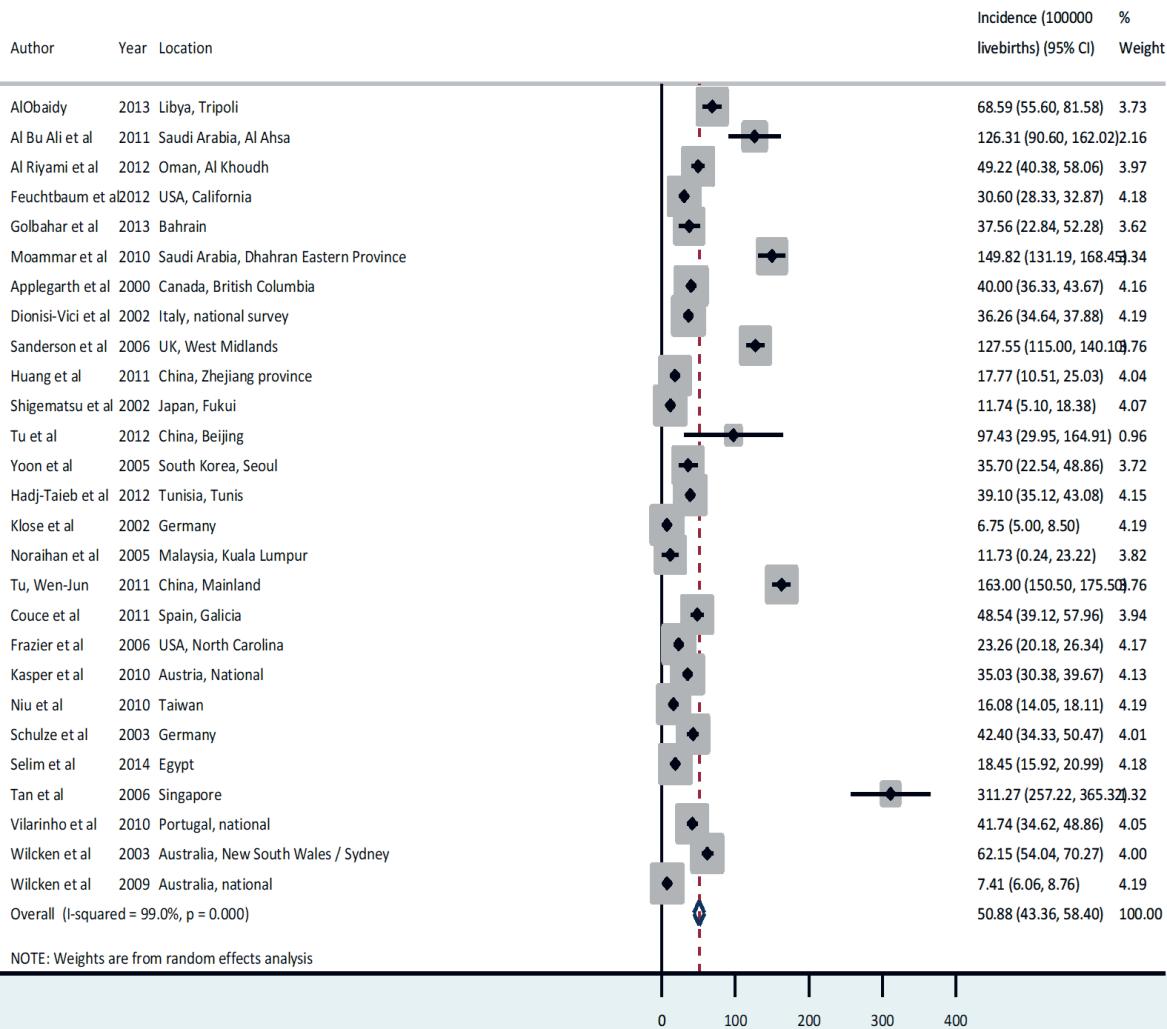
Birth prevalence of all inborn errors of metabolism (IEM).

**Figure 2** shows results for the meta-analysis of all IEM, with an overall birth prevalence estimate of 50.9 per 100 000 live births (live births) (95% confidence intervals 43.4-58.4 per 100 000 LB).

**Figure 3:**
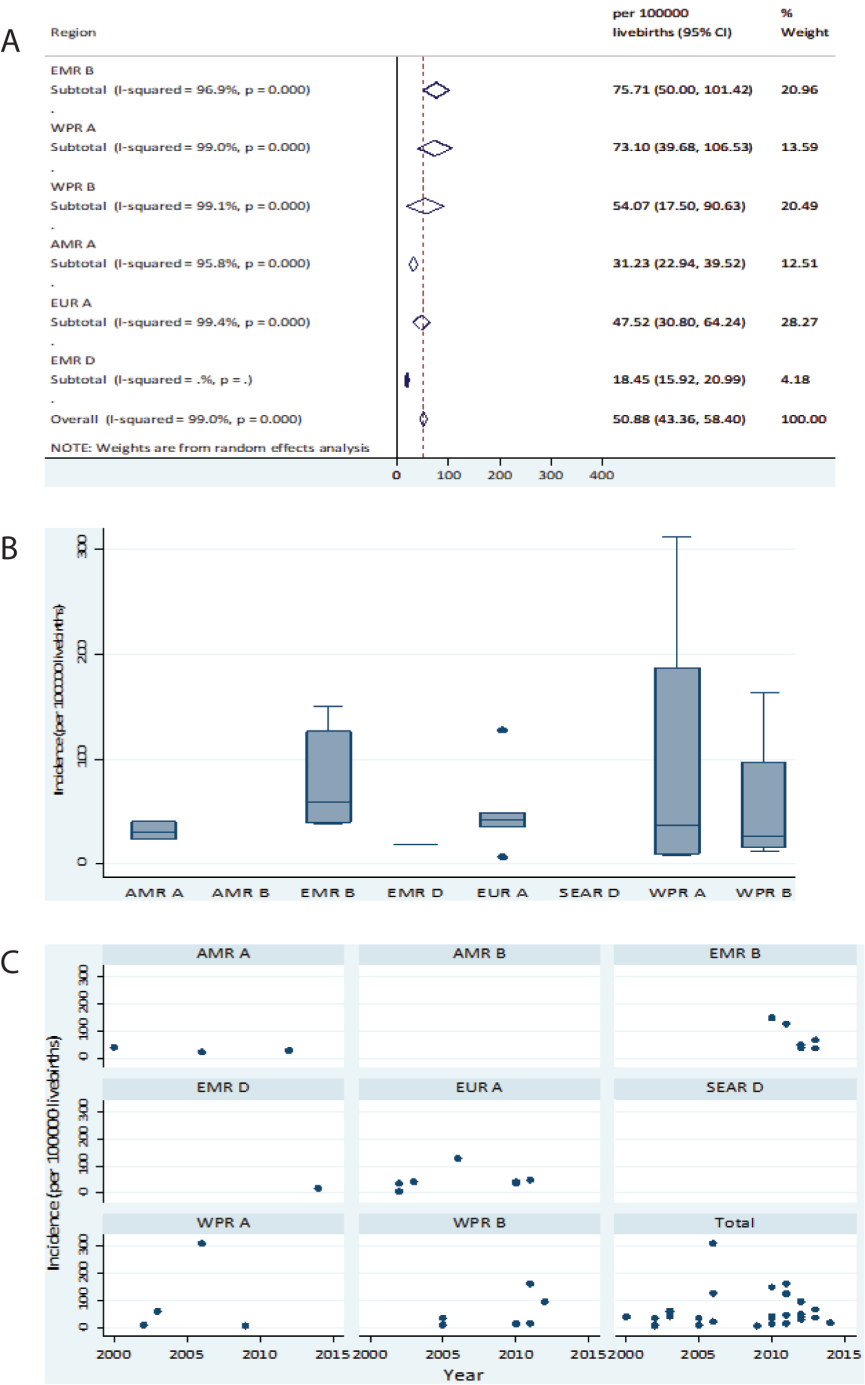
**A.** Regional meta-estimate of birth prevalence of all inborn errors of metabolism (IEM). **B**. Regional birth prevalence rates distribution of all IEM. **C.** Birth prevalence of IEM from all causes according to study region and period.

From the regional meta-estimates, ***EMR B*** and ***WPR A*** had the highest birth prevalence rates of all-cause IEM at 75.7 (95% CI = 50.0-101.4) and 73.1 (95% CI = 39.7-106.5) per 100 000 live births (LB) respectively (**Figure 3**, plates A-C).

Meta-estimates of individual classes of IEM showed amino acidurias to have the highest birth prevalence with 14.7 cases (95% CI = 12.2-17.2) per 100 000 LB, followed by lysosomal storage disorders with 13.3 (95% CI = 9.48-17.02) per 10 000 LB. Other IEM classes that reported high birth prevalence included organic acidurias, mitochondrial disorders, fatty acidurias and carbohydrate metabolism disorders with 8.7 (95% CI = 7.2-10.3), 8.2 (95% CI = 5.4-10.9), 6.5 (95% CI = 5.2-7.9) and 6.2 (95% CI = 4.5-7.9) cases per 100 000 LB, respectively. Some other notable IEM subclasses include phenylketonuria (a variant of amino aciduria) and medium chain acyl-coenzyme A dehydrogenase deficiency (a variant of fatty aciduria) with 6.6 (95% CI = 5.3-7.8) and 5.8 (95% CI = 4.4-7.2) per 100 000 LB respectively (45% of total amino acidurias and 89% of total fatty acidurias respectively) .When the crude estimates of the individual eight classes included in this review were pooled together (sensitivity analysis), we recorded a pooled estimate of 64.6 (95% CI = 48.5-81.1) cases per 100 000 LB (**Table 4** & Appendix 3 in **Online Supplementary Document(Online Supplementary Document)**).

**Table 4:** Birth prevalence of all and individual classes of inborn errors of metabolism (IEM) globally

IEM	Class	Birth prevalence (per 100 000 LB)	Confidence interval	Heterogeneity
All IEM	All	50.88	43.36-58.4	I-squared = 99.0%, *P* < 0.001
Amino acid disorders	All	14.69	12.20-17.17	I-squared = 97.8%, *P* < 0.001
	Phenylketonuria	6.55	5.34-7.76	I-squared = 95.7%, *P* < 0.001
	Maple syrup urine disease	1.22	0.82-1.61	I-squared = 87.3%, *P* < 0.001
	Homocystinuria	0.41	0.20-0.63	I-squared = 81.7%, *P* < 0.001
Organic acid disorders	All	8.71	7.15-10.27	I-squared = 93.5%, *P* < 0.001
	Propionic aciduria	1.07	0.73-1.42	I-squared = 90.5%, *P* < 0.001
	Methyl malonic aciduria	1.68	1.19-2.16	I-squared = 89.3%, *P* < 0.001
	Isovaleric aciduria	0.51	0.28-0.73	I-squared = 68.1%, *P* < 0.001
	Biotinidase deficiency	1.64	1.02-2.26	I-squared = 90.5%, *P* < 0.001
Fatty acid disorders	All	6.51	5.14-7.89	I-squared = 96.2%, *P* < 0.001
	MCAD deficiency	5.78	4.38-7.18	I-squared = 96.8%, *P* < 0.001
Lysosomal storage disorders	All	13.25	9.48-17.02	I-squared = 98.6%, *P* < 0.001
Carbohydrate metabolism disorders	All	6.19	4.45-7.94	I-squared = 71.6%, *P* = 0.002
Urea cycle disorders	All	2.91	1.90-3.92	I-squared = 83.7%, *P* < 0.001
Mitochondrial disorders	All	8.16	5.42-10.91	I-squared = 92.2%, *P* < 0.001
Peroxisomal disorders	All	4.13	2.78-5.48	I-squared = 95.4%, *P* < 0.001

MCAD – medium chain acyl-coenzyme A dehydrogenase

### Parental consanguinity rates

**Figure 4:**
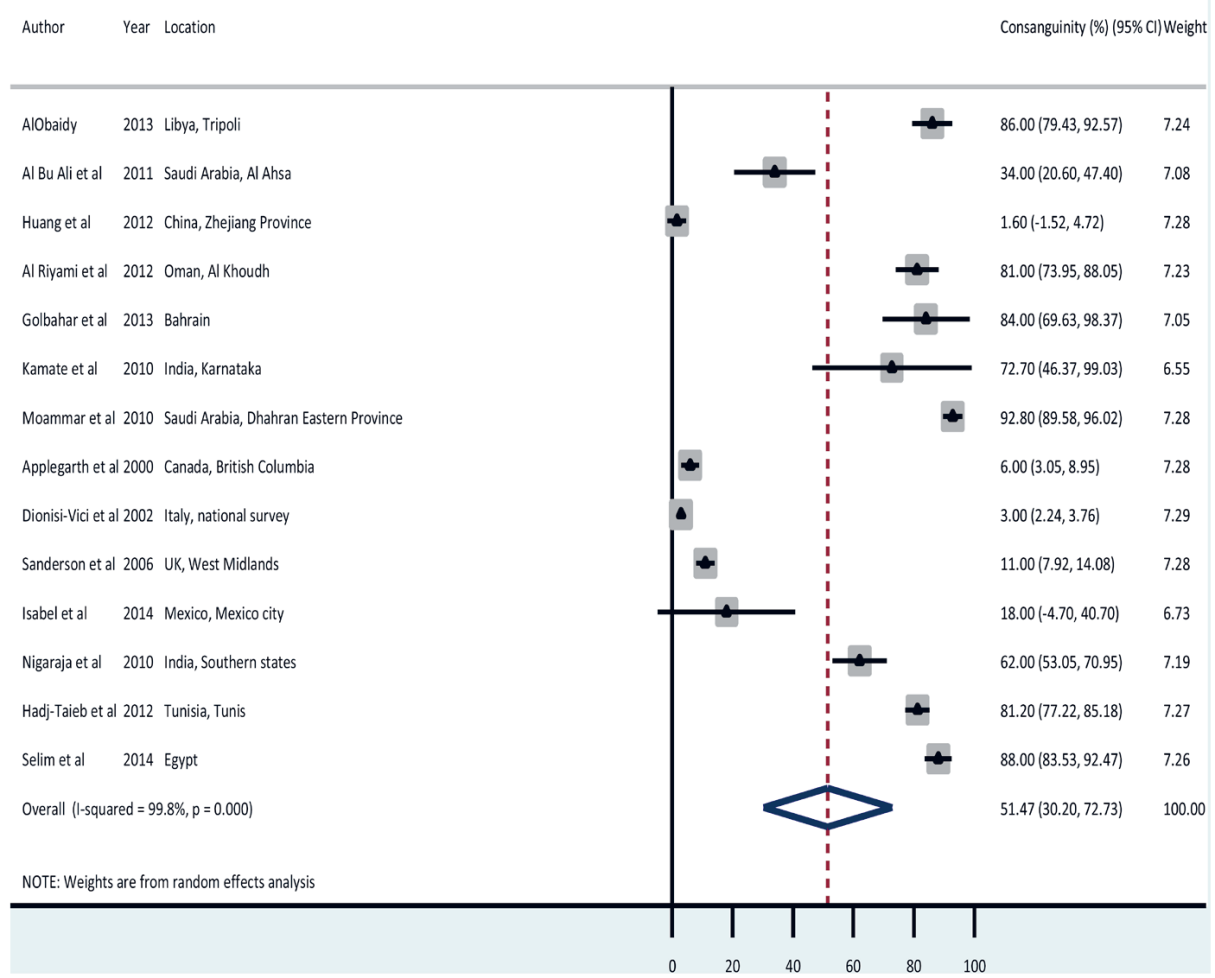
Meta-estimate of consanguinity rates of inborn errors of metabolism (IEM) from all causes.

Few studies (n = 14) reported parental consanguinity rates among cases of IEM. The overall meta-estimate was 51.47 (95% CI = 30.20-72.73) percent (shown in **Figure 4**). From individual studies, Saudi Arabia, Egypt, Libya, Bahrain, Tunisia and India had the highest consanguinity rates at 92%, 88%, 86%, 84%, 81% and 73% respectively. Lowest rates were recorded in China (1.6%), Italy (3%), Canada (6%) and the United Kingdom (11%).

### Deaths attributable to IEM

Data on CFR were not available from the majority of studies, **Table 5** shows data from studies that did describe CFR (n = 7). For all studies, data were reported with a denominator of life births and the length of follow-up of the birth cohort being studied was unclear, resultantly it was not possible to determine the age or age group at which deaths occurred.

**Table 5:** Deaths attributable to inborn errors of metabolism (IEM)

Author	Year	Location	Setting	% consanguinity	Total LB	IEM cases	Birth prev/ 10^5^ LB	Deaths	Deaths/10^5^ LB	Case fatality rate (%)
AlObaidy et al [13]	2013	Libya, Tripoli	Urban	86	156006	107	68.6	33	22.4	32.7
Dionisi-Vici et al [23]	2002	Italy, national survey	Urban-Rural	3	5336730	1935	36.3	356	6.4	18.4
Klose et al [32]	2002	Germany	Urban-Rural	N/A	844575	57	6.8	8	1.0	14.0
Couce et al [35]	2011	Spain, Galicia	Urban	N/A	210165	137	65.2	4	1.9	2.9
Frazier et al [36]	2006	USA, North Carolina	Urban	N/A	944078	219	23.3	7	0.7	3.2
Schulze et al [8]	2003	Germany	Urban-Rural	N/A	250000	106	42.4	3	1.2	2.8
Wilcken et al [42]	2009	Australia, national	Urban-Rural	N/A	1551200	115	7.4	26	1.7	22.6

N/A – not applicable, LB – live birth(s)

**Figure 5:**
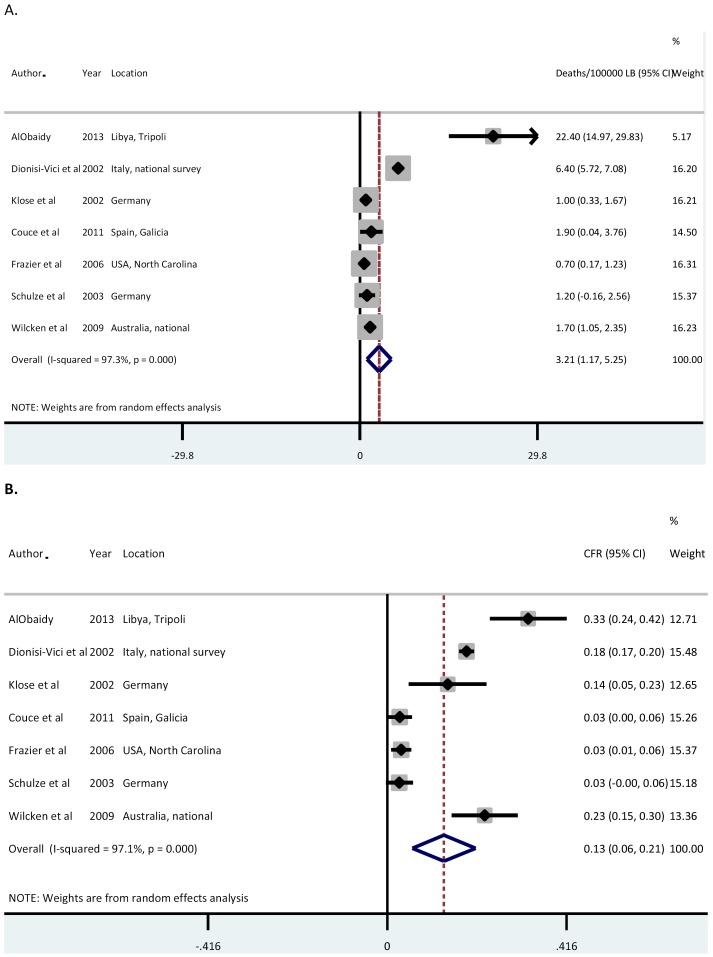
**A.** Meta-estimate of deaths attributable to inborn errors of metabolism (IEM) globally. **B.** Meta-estimate of case fatality rates from IEM globally.

Our overall meta-estimate of deaths attributable to IEM globally is 3.2 (95% CI = 1.2-5.3) per 100 000 LB, with a corresponding CFR of 13% (95% CI = 6%21%) (**Figure 5**, panels A and B). AlObaidy et al reported in Libya a CFR over 30 times higher than Frazier et al in the USA (22.4 per 100 000 LB compared with 0.7 per 100 000 LB), likely in part due to differences in diagnosis, management and overall health care between and middle- and high-income settings [13,36].

## DISCUSSION

### Main findings

This study, as far as we are aware, provides the first global estimates of the birth prevalence and mortality from IEM among children under 5 years of age, in an effort to increase understanding of these potentially important causes of global child morbidity and mortality.

The estimated birth prevalence of all IEM globally was 50.9 (95% CI = 43.4-58.4) per 100 000 LB. This falls within the confidence intervals of the pooled birth prevalence rates of the eight classes of IEM included in this review (shown in **Table 4**) of 64.6 cases per 100 000 LB (95% CI = 48.5-81.1), adding confidence to our overall estimate (see also Appendix 3 in **Online Supplementary Document(Online Supplementary Document)**). Our estimate of all IEM globally implies 500 IEM cases for every 1 million live births globally, suggesting 70 587 (95% CI = 61 147-82 281) new cases of IEM per year, based on UNDP projections for live births in the period 2015-2020 [43]. This estimate may not be representative of all world regions, as it does not include data from all regions and income groups, or all possible IEM classes. In addition IEM are highly susceptible to underestimation due to missed diagnosis (a factor we were unable to account for). It is therefore likely our figure represents an under-estimate.

Regional pooled birth prevalence rates showed that ***EMR B*** had the highest IEM birth prevalence rate of all-cause IEM at 75.7 (95% CI = 50.0-101.4) per 100 000. Although this could be a result of selection bias, it may also reflect this as an area with higher parental consanguinity rates which would be expected to report increased IEM birth prevalence [7,44]. Studies from the EMR B region showed the highest parental consanguinity rates in IEM cases of 34%-93% compared with the overall pooled consanguinity rate of 51.47% (95% CI = 30.20-72.73%).

The estimated global deaths attributable to all-cause IEM were 3.2 (1.2-5.3) per 100 000 LB, with a meta estimate of CFR of 13% (95% CI = 6-21%). Again, these figures are likely to reflect an under-estimation as all but one study used for these estimates came from high-income countries, and none came from low-income countries. It should also be emphasised many early childhood deaths from IEM occur without an accurate diagnosis, particularly in LMICs [4,5]. Our data suggest a CFR of IEM in high-income settings ranging from 2%-23%, rising to 33% in middle-income countries, and possibly higher still in low-income countries (**Table 5**). Given that the vast majority of all IEM cases will occur in LMICs it may be appropriate to assume that 33% represents a low bound of possible estimate for overall global CFR. This CFR applied to our estimated birth prevalence and combined with UNPD projections would suggest 23 529 deaths per year from IEM (95% CI = 20 382-27 427), approximating to 0.4% of all child deaths globally [1,43].

### Limitations

There are several important limitations to the conclusions of this study. Of the 49 studies selected for this review, only 33 studies provided estimate on all-cause IEM. These studies were focused in certain geographical areas, with no studies from low-income countries. The lack of studies from sub-Saharan Africa is of particular concern as this region has the highest number of under 5 deaths globally [1]. In addition, only seven studies were birth cohort studies (with reference denominators provided), limiting the representativeness of our conclusions due to potential selection bias. It was unfortunate that no included studies reported clear follow-up periods, which could have allowed a more meaningful interpretation of mortality and CFR over the first five years of life. Additionally, some important studies that could have improved the quality of this review were conducted on children aged 0-14 years, without any breakdown of estimates by age, meaning that relevant estimates in 0-4 years could not be extracted. It is also important to note that many studies were conducted in areas with high consanguinity rates, likely to result in a higher IEM prevalence than the overall global population [7,44].

### Findings in context

This study highlights the need for better understanding of IEM globally to facilitate improved diagnosis and management, particularly in sub-Saharan Africa where no data are currently available. Further large-scale birth cohort studies are necessary to improve knowledge on the epidemiology of this important group of conditions.

Despite data limitations, our study has shown IEM represent an important cause of under 5 morbidity and mortality. Outcomes can be markedly improved by early recognition of IEM, and so a focus on increased clinician awareness globally and improved access to necessary investigations is essential to avoid missed diagnoses [2]. Newborn screening for common IEM has proved highly effective in many settings and it is important that capacity is built in LMIC health systems to foster similar screening programmes [9,45]. Laboratory capacity may be a significant limiting factor in screening and other investigations, particularly for novel and more complex metabolomic diagnostics, and we emphasise this as an important area to focus on with regards to health system strengthening [3,6,45,46].

Our data also reinforces the fact that parental consanguinity plays an important role in the incidence of IEM, focused particularly in certain areas of the world [7,44]. Although often socioculturally complex, interventions to tackle this at a community level could play an important role in decreasing the number of births with IEM globally [47].

Treatment approaches for IEM are another essential area of focus. Emerging therapeutics including the use of molecular chaperones and substrate synthesis inhibitors show promising initial results, however due to high cost, are likely to be out of reach of many areas globally for the foreseeable future [48,49]. Despite this, the potential for extending existing management strategies employed in high-income settings to lower resourced populations has much potential to impact on global IEM morbidity and mortality [50]. In LMICs where access to paediatric endocrinology expertise is often limited or non-existent, even with an accurate diagnosis of an IEM, appropriate management can be difficult to provide [50,51]. The development and propagation of clear national and international protocols for managing IEM and associated metabolic crises could provide one avenue of improving the standard of care for this important patient group.

## CONCLUSIONS

IEM represent an important cause of global child morbidity and mortality, comprising a significant proportion of child deaths which are as of yet poorly described in global modelling efforts. Although more population-level research is required to further establish the epidemiology of these conditions, our study emphasises several areas for current policy focus, including enhanced laboratory capacity for screening and diagnosis, community interventions to tackle parental consanguinity, and dissemination of knowledge regarding awareness and management of these important conditions.
