# Supplementary material for: Global birth prevalence and mortality from inborn errors of metabolism: a systematic analysis of the evidence
Source: J Glob Health. 2018 Nov 14;8(2):021102. doi: 10.7189/jogh.08.021102 (PMC6237105; doi:10.7189/jogh.08.021102)
Supplement: Online Supplementary Document [file jogh-08-021102-s001.pdf]

Online Supplementary Document

Waters et al. Global birth prevalence and mortality from inborn errors of metabolism.

J Glob Health 2018;8:021102

Appendix 1: Search terms used for literature search

A 1.1: Search terms used for literature search (MEDLINE)

#	Searches	Hits
**1**	exp vital statistics/ or exp incidence/	689447
**2**	(incidence* or prevalence* or morbidity or mortality).tw.	1296484
**3**	(disease adj3 burden).tw.	11981
**4**	exp "cost of illness"/	18825
**5**	exp quality-adjusted life years/	7483
**6**	QALY.tw.	4182
**7**	Disability adjusted life years.mp.	983
**8**	(initial adj2 burden).tw.	194
**9**	exp risk factors/	593406
**10**	1 or 2 or 3 or 4 or 5 or 6 or 7 or 8 or 9	1990285
**11**	metabolism, inborn errors/ or exp amino acid metabolism, inborn errors/ or exp amino acid transport disorders, inborn/ or exp amyloidosis, familial/ or exp brain diseases, metabolic, inborn/ or exp carbohydrate metabolism, inborn errors/ or exp hyperbilirubinemia, hereditary/ or exp lipid metabolism, inborn errors/ or exp lysosomal storage diseases/ or exp metal metabolism, inborn errors/ or exp peroxisomal disorders/ or exp porphyrias/ or exp purine-pyrimidine metabolism, inborn errors/ or exp renal tubular transport, inborn errors/ or exp steroid metabolism, inborn errors/	144169
**12**	congenital metabolic disorders.mp. [mp=title, abstract, original title, name of substance word, subject heading word, keyword heading word, protocol supplementary concept word, rare disease supplementary concept word, unique identifier]	77
**13**	inherited metabolic disorders.mp. [mp=title, abstract, original title, name of substance word, subject heading word, keyword heading word, protocol supplementary concept word, rare disease supplementary concept word, unique identifier]	405
**14**	11 or 12 or 13	144323
**15**	10 and 14	12761
**16**	**limit 15 to (yr="1980 -Current" and "all child (0 to 18 years)")**	4872

A 1.2: Search terms used for literature search (EMBASE)

#	Searches	Hits
**1**	exp vital statistics/ or exp incidence/	282502
**2**	(incidence* or prevalence* or morbidity or mortality).tw.	1831362
**3**	(disease adj3 burden).tw.	19994
**4**	exp "cost of illness"/	15304
**5**	exp quality-adjusted life years/	13770
**6**	QALY.tw.	8215
**7**	Disability adjusted life years.mp.	1466
**8**	(initial adj2 burden).tw.	291
**9**	exp risk factors/	673443
**10**	1 or 2 or 3 or 4 or 5 or 6 or 7 or 8 or 9	2393629
**11**	metabolism, inborn errors/ or exp amino acid metabolism, inborn errors/ or exp amino acid transport disorders, inborn/ or exp amyloidosis, familial/ or exp brain diseases, metabolic, inborn/ or exp carbohydrate metabolism, inborn errors/ or exp hyperbilirubinemia, hereditary/ or exp lipid metabolism, inborn errors/ or exp lysosomal storage diseases/ or exp metal metabolism, inborn errors/ or exp peroxisomal disorders/ or exp porphyrias/ or exp purine-pyrimidine metabolism, inborn errors/ or exp renal tubular transport, inborn errors/ or exp steroid metabolism, inborn errors/	1161746
**12**	congenital metabolic disorders.mp. [mp=title, abstract, subject headings, heading word, drug trade name, original title, device manufacturer, drug manufacturer, device trade name, keyword]	93
**13**	inherited metabolic disorders.mp. [mp=title, abstract, subject headings, heading word, drug trade name, original title, device manufacturer, drug manufacturer, device trade name, keyword]	659
**14**	11 or 12 or 13	1161948
**15**	10 and 14	261090
**16**	limit 15 to (yr="1980 -Current" and child <unspecified age>)	17130

A 1.3: Search terms used for literature search (Global Health)

#	Searches	Hits
**1**	exp vital statistics/ or exp incidence/	121400
**2**	(incidence* or prevalence* or morbidity or mortality).tw.	391951
**3**	(disease adj3 burden).tw.	6599
**4**	exp "cost of illness"/	0
**5**	exp quality-adjusted life years/	0
**6**	QALY.tw.	799
**7**	Disability adjusted life years.mp.	830
**8**	(initial adj2 burden).tw.	24
**9**	exp risk factors/	132753
**10**	1 or 2 or 3 or 4 or 5 or 6 or 7 or 8 or 9	470049
**11**	congenital metabolic disorders.mp. [mp=abstract, title, original title, broad terms, heading words, identifiers, cabicodes]	12
**12**	inherited metabolic disorders.mp. [mp=abstract, title, original title, broad terms, heading words, identifiers, cabicodes]	79
**13**	exp congenital metabolic anomalies/ or exp metabolic disorders/	82264
**14**	11 or 12 or 13	82288
**15**	10 and 14	29353
**16**	limit 15 to yr="1980 -Current"	29168
**17**	exp children/	185225
**18**	16 and 17	3034

Appendix 2. Quality grading

For the quality grading, a modified Grading of Recommendations Assessment, Development and Evaluation (GRADE) guidelines was adapted. Studies were graded as follows:

- High quality: Studies with the entire four criteria, or any three including “study design”, highlighted above well represented;
- Moderate quality: Studies with any three of the four criteria, or any two including “study design”, highlighted above well represented;
- Low quality: Studies with any two of the four criteria, or “study design” only, highlighted above well represented; and
- Very low quality: Studies with only one (excluding “study design”) or none of the four criteria highlighted above well represented.

Appendix 3: Meta-estimates of specific IEM

A 3.1. Meta-estimate of incidence of all amino acid disorders

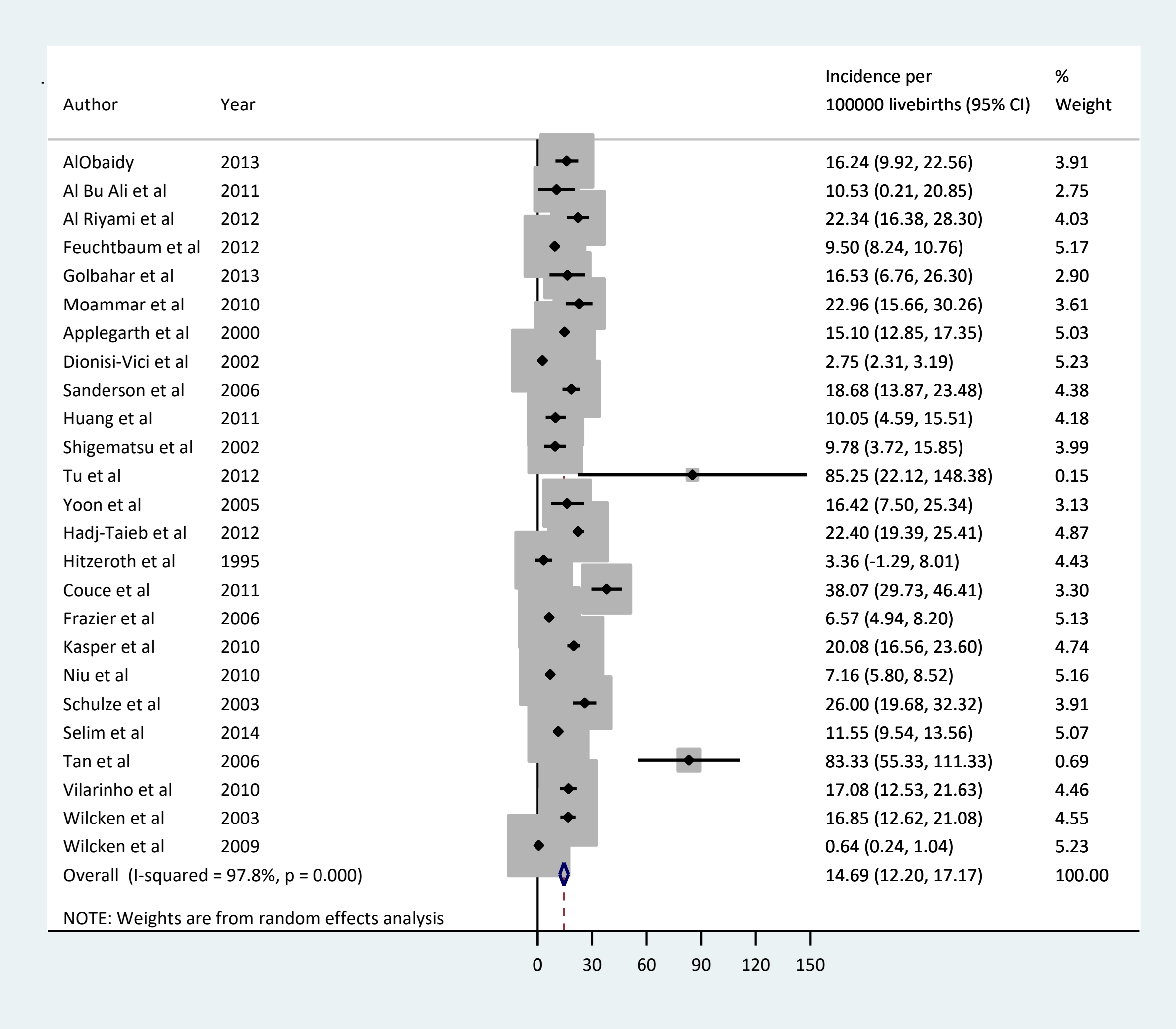

A 3.2. Meta-estimate of incidence of phenylketonuria

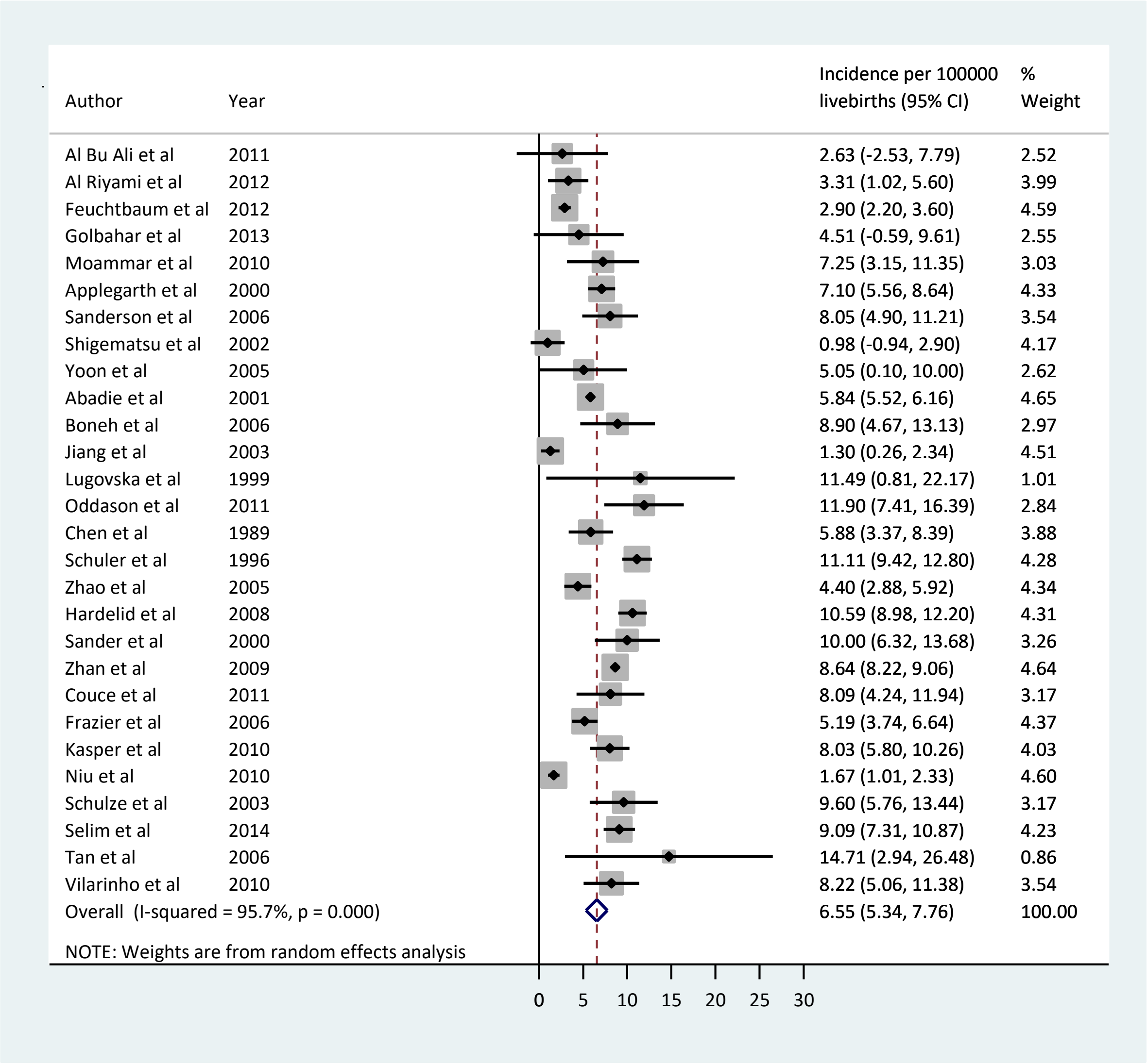

A 3.3. Meta-estimate of incidence of maple syrup urine disease

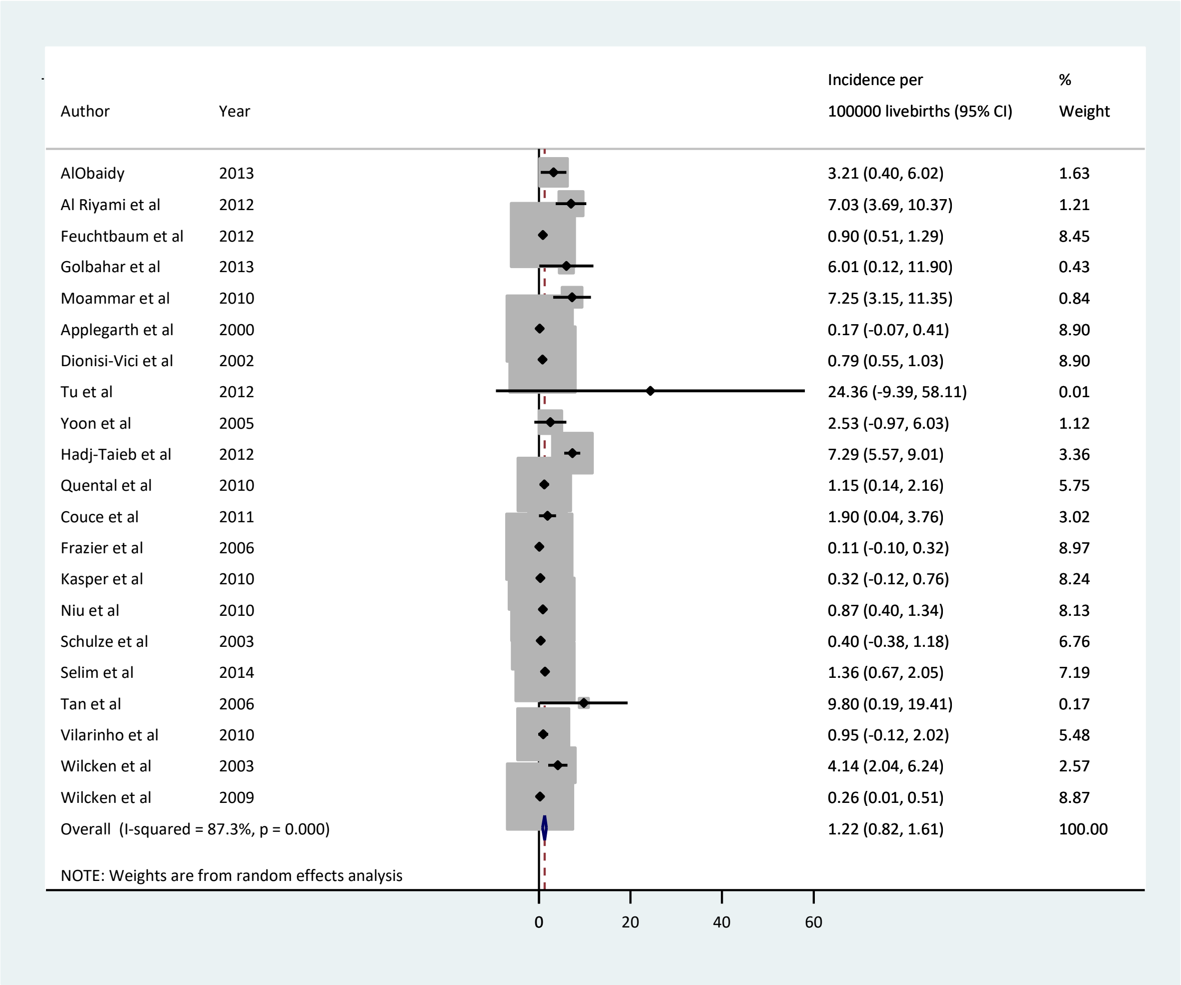

A 3.4. Meta-estimate of incidence of homocystinuria

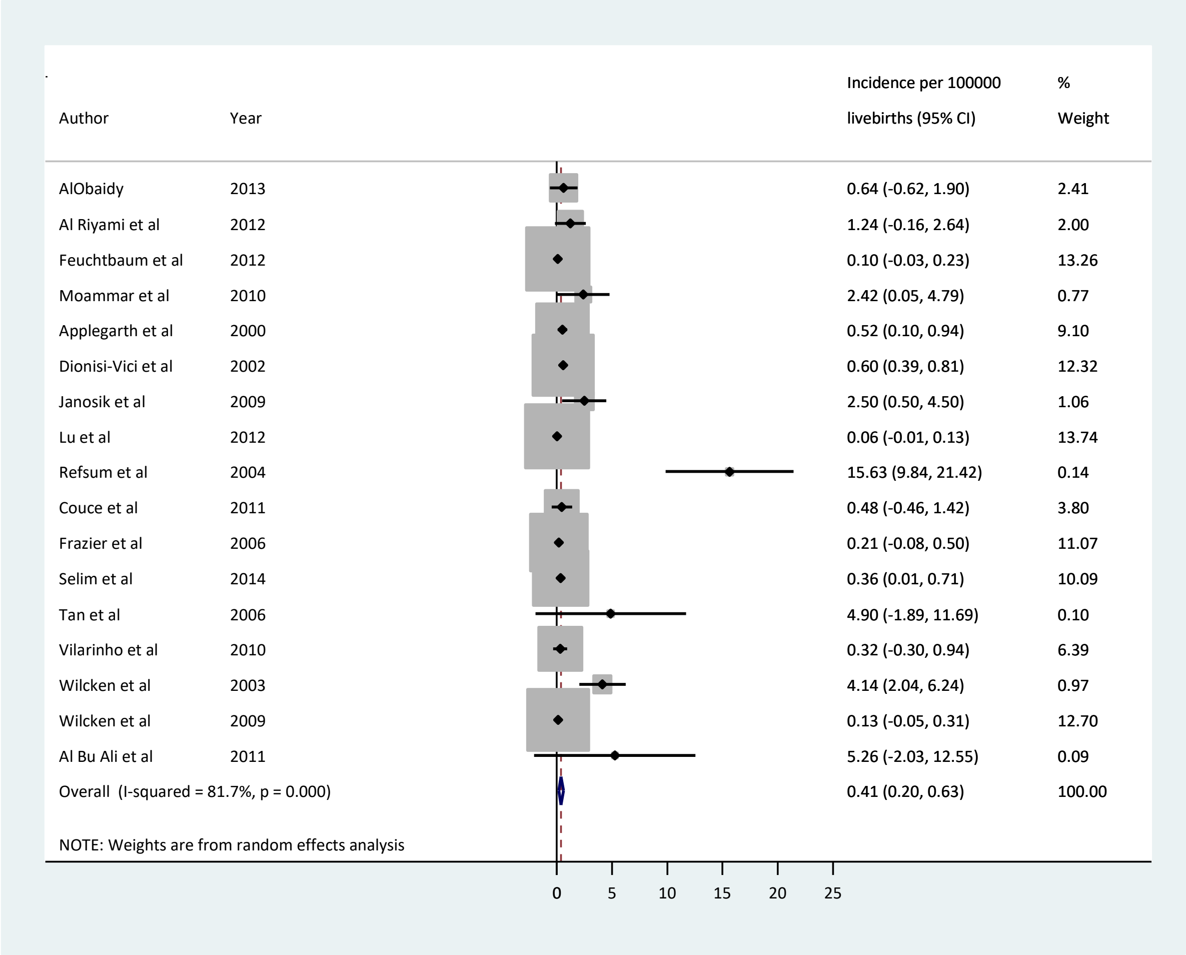

A 3.5. Meta-estimate of incidence of all organic acidurias

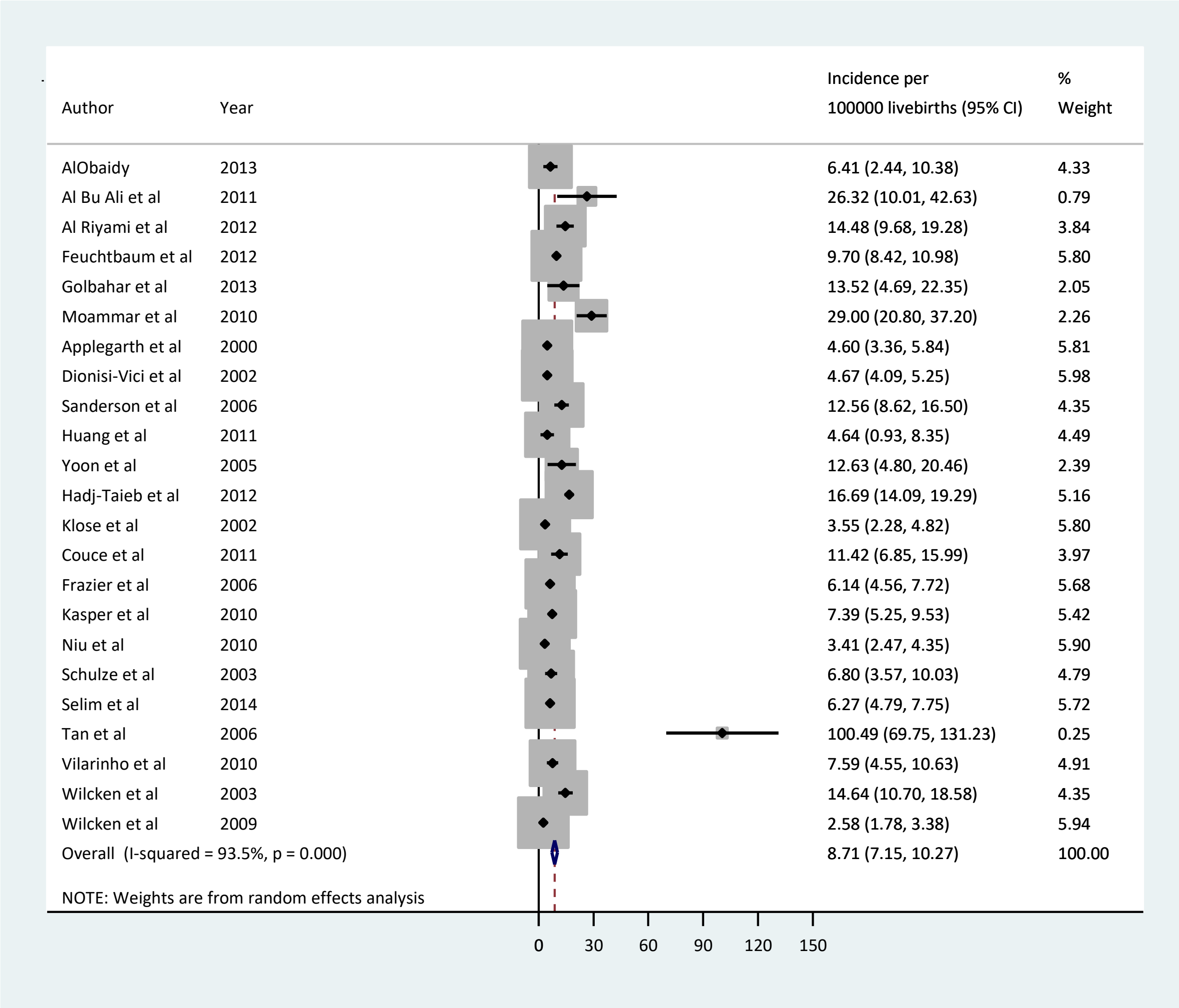

A 3.6. Meta-estimate of incidence of propionic aciduria

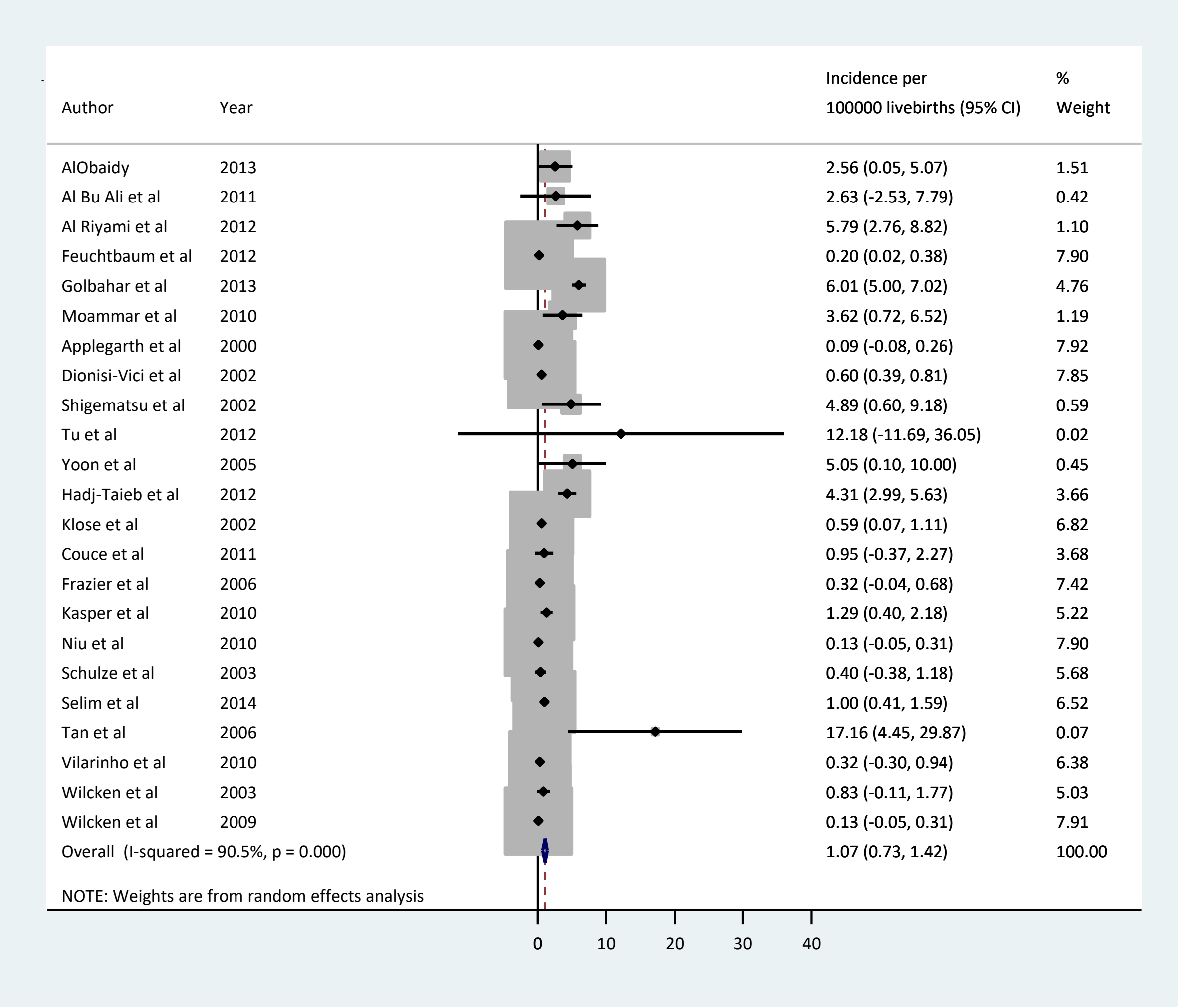

A 3.7. Meta-estimate of incidence of methyl malonic aciduria

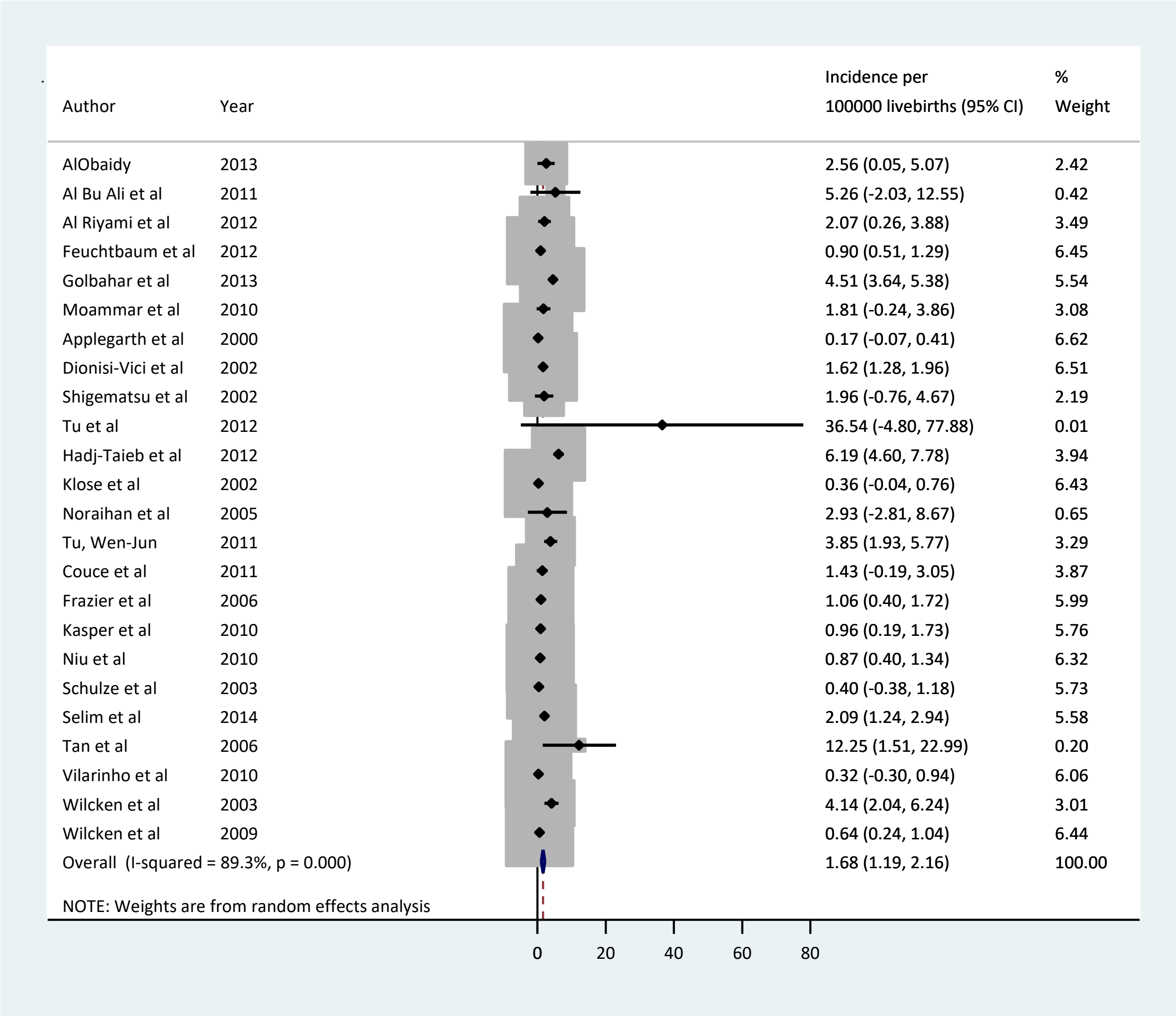

A 3.8. Meta-estimate of incidence of isovaleric aciduria

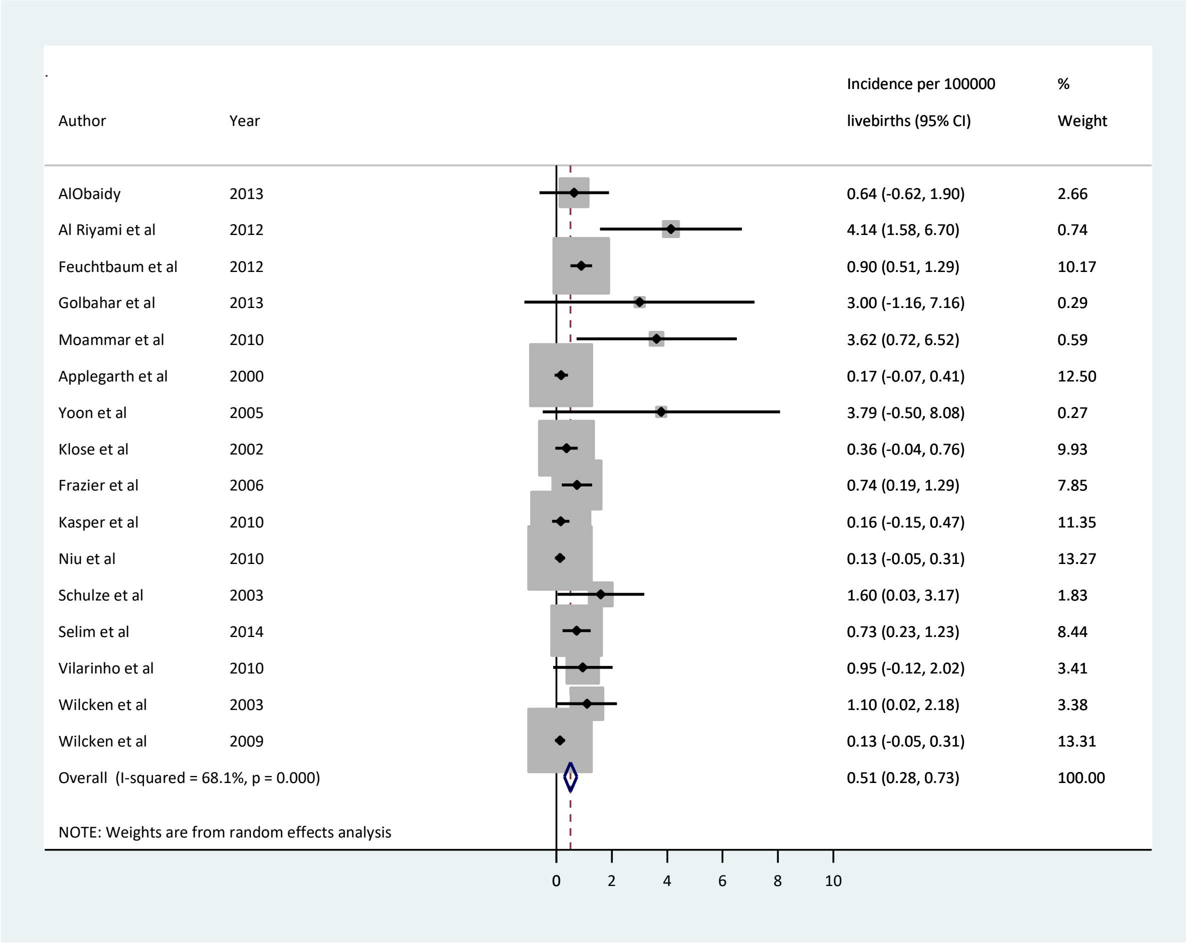

A 3.9. Meta-estimate of incidence of biotinidase deficiency

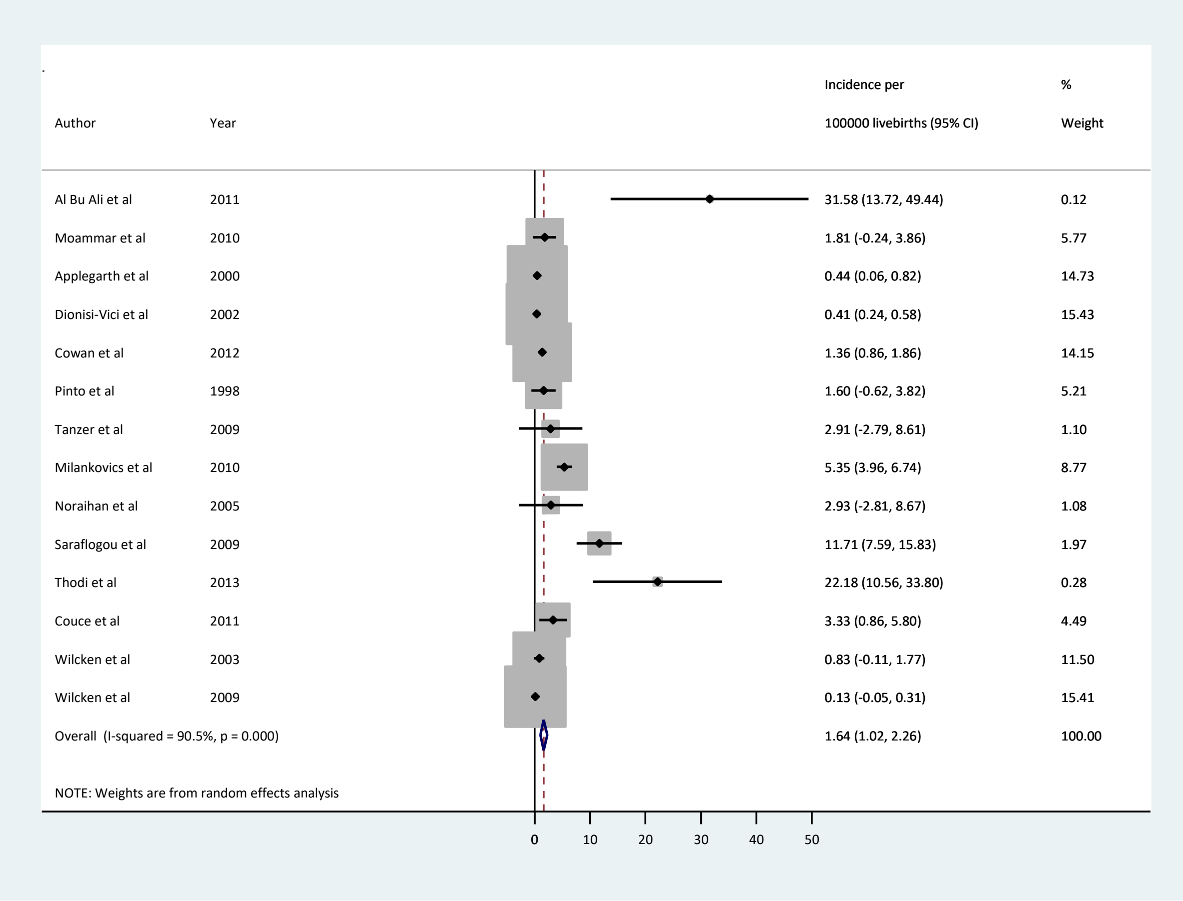

A 3.10. Meta-estimate of incidence of all fatty acid disorders

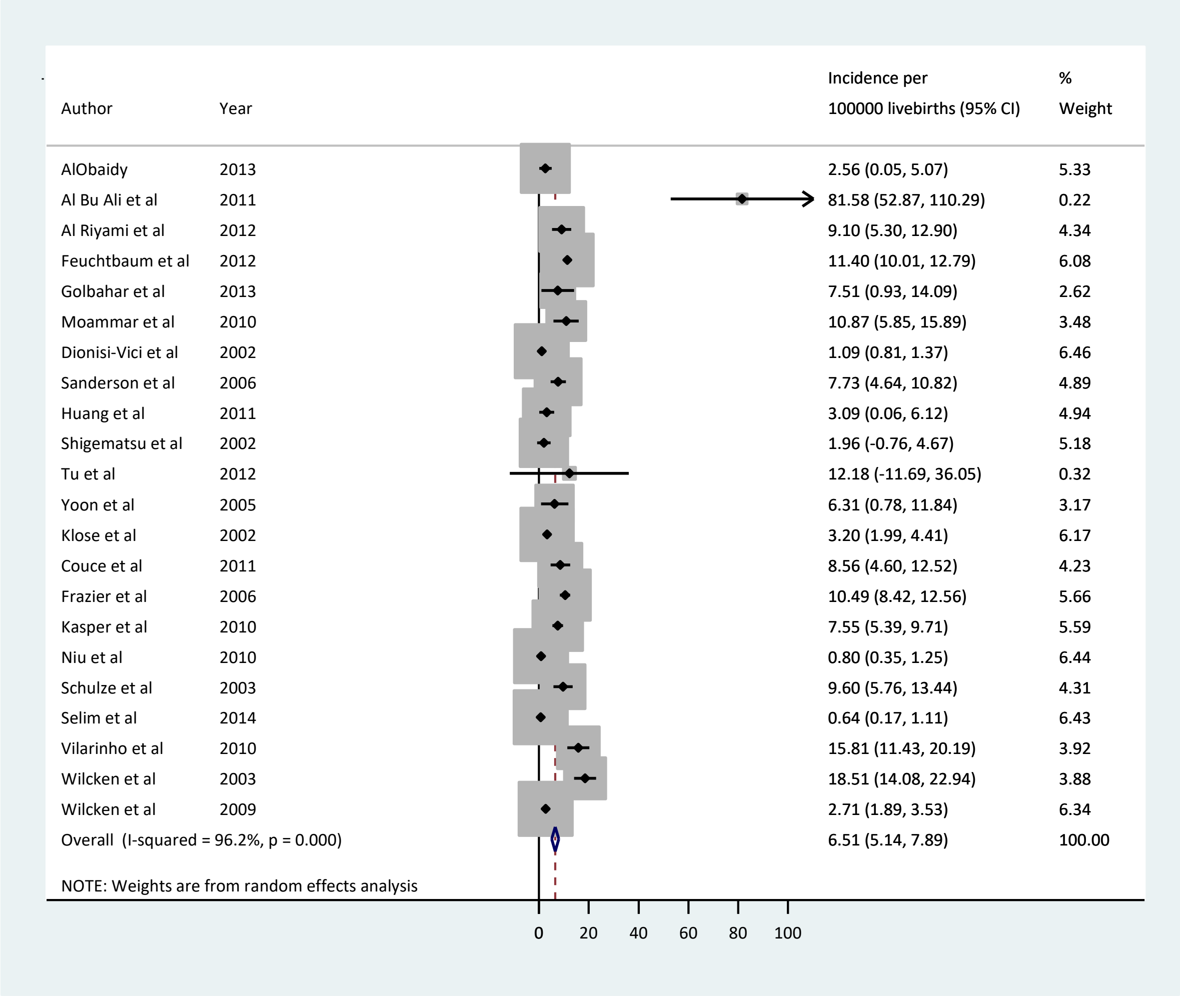

A 3.11. Meta-estimate of incidence of medium chain acyl coenzyme A dehydrogenase (MCAD) deficiency

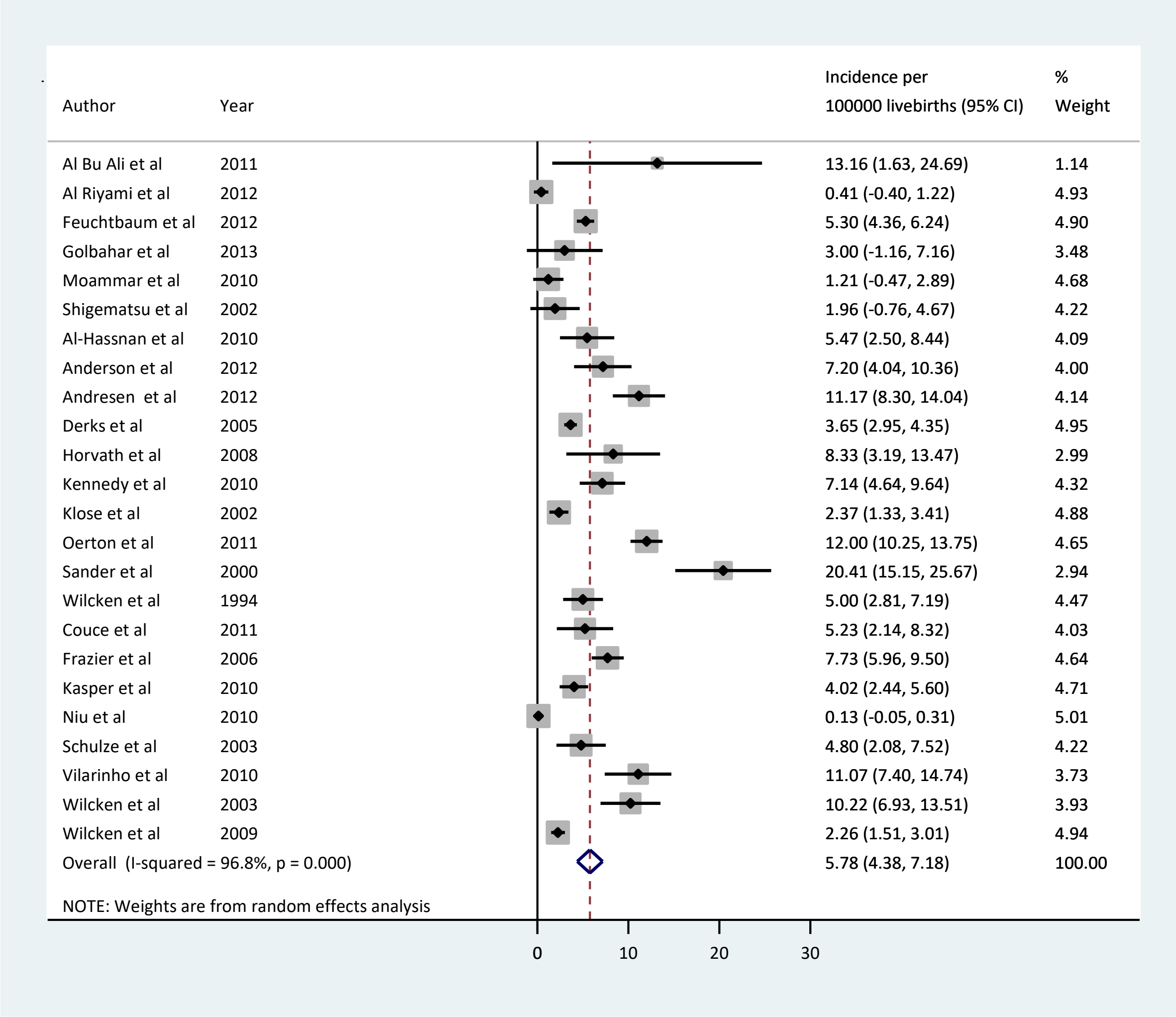

A 3.12. Meta-estimate of incidence of all lysosomal storage disorders

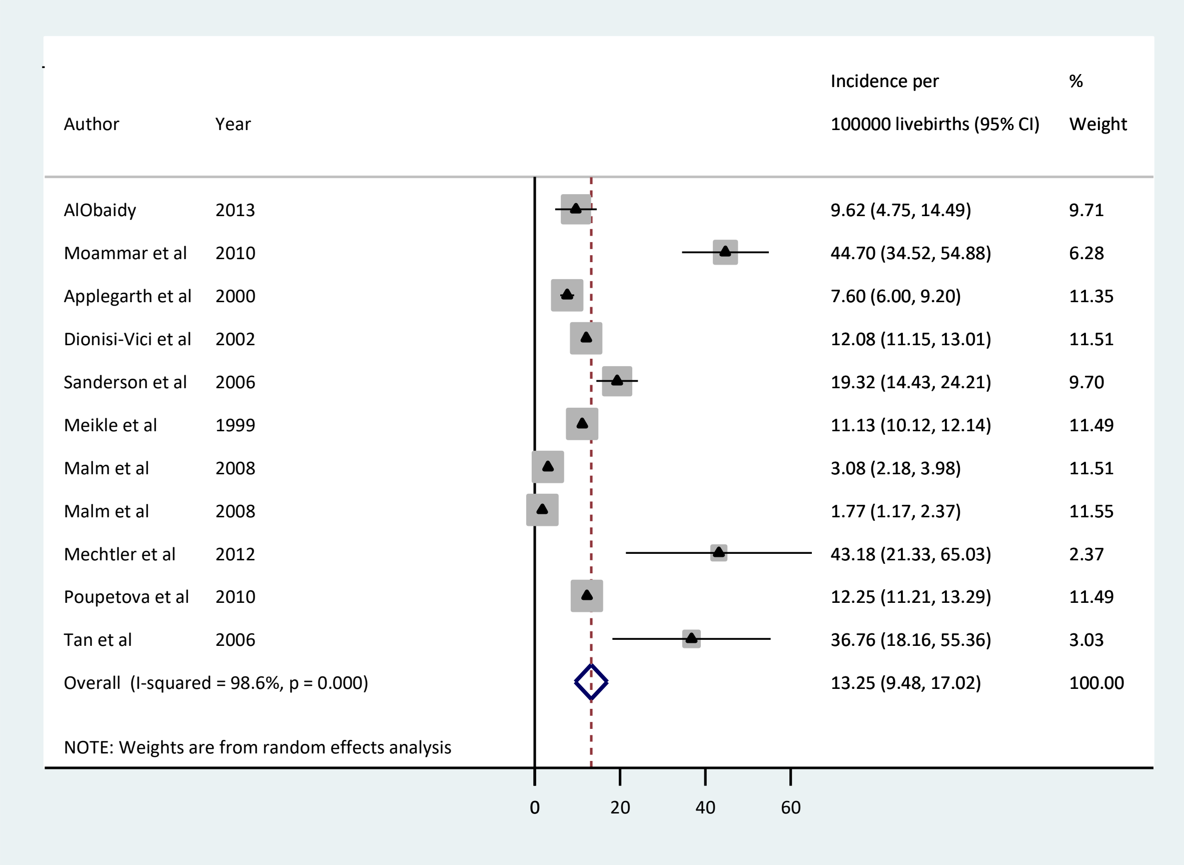

A 3.13. Meta-estimate of incidence of all carbohydrate metabolism disorders

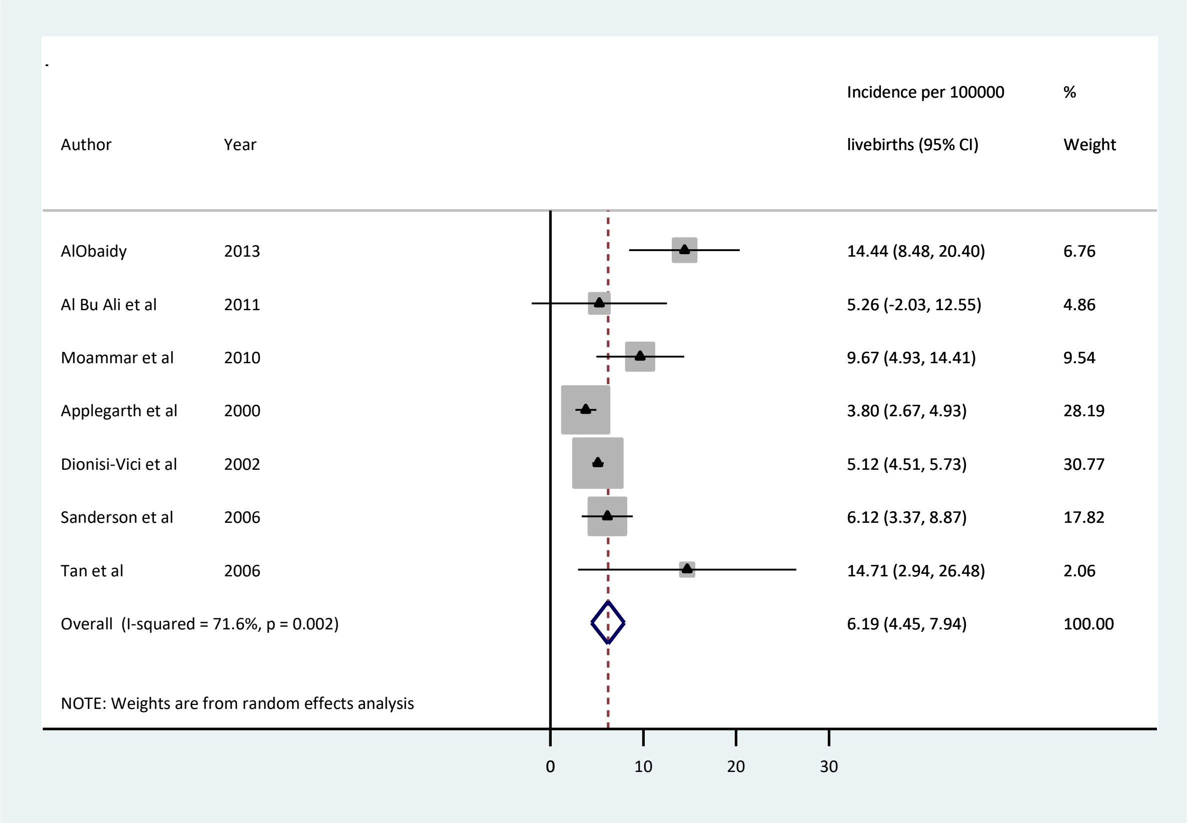

A 3.14. Meta-estimate of incidence of all urea cycle disorders

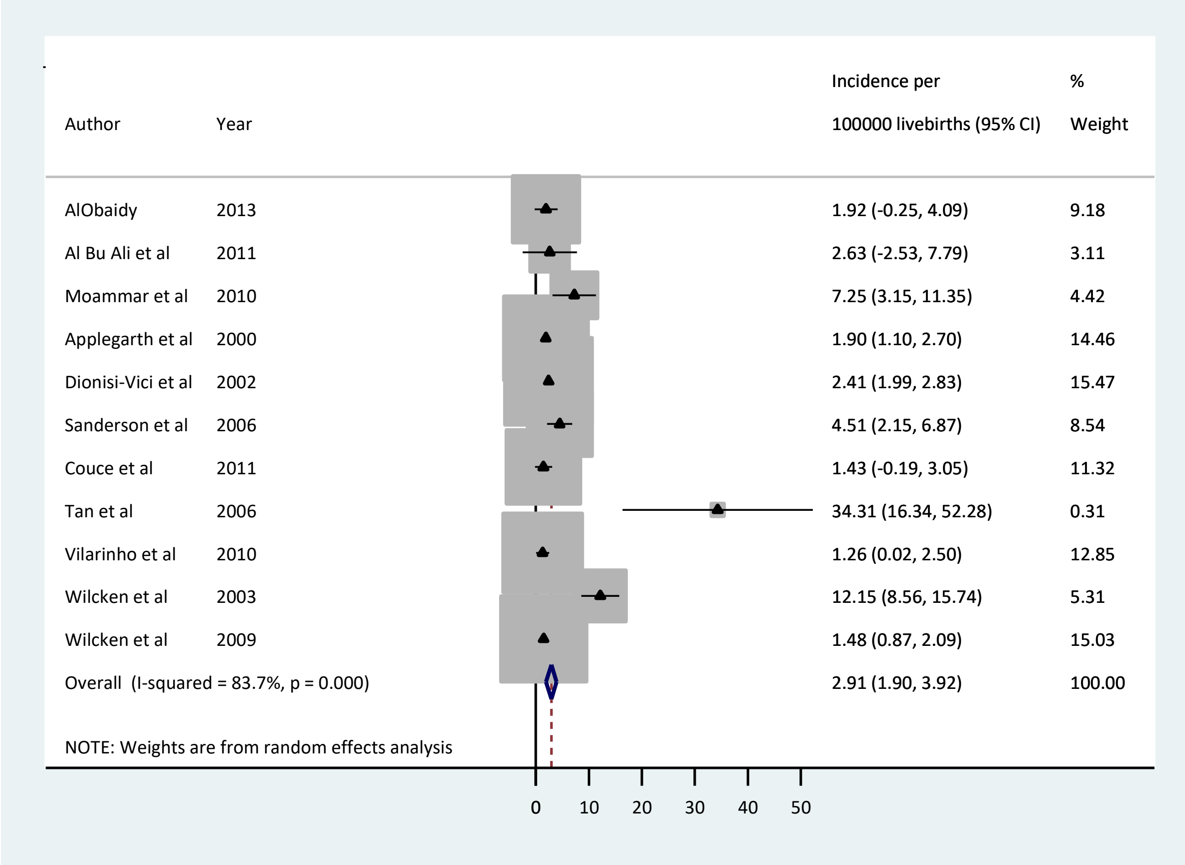

A 3.15. Meta-estimate of incidence of all mitochondrial disorders

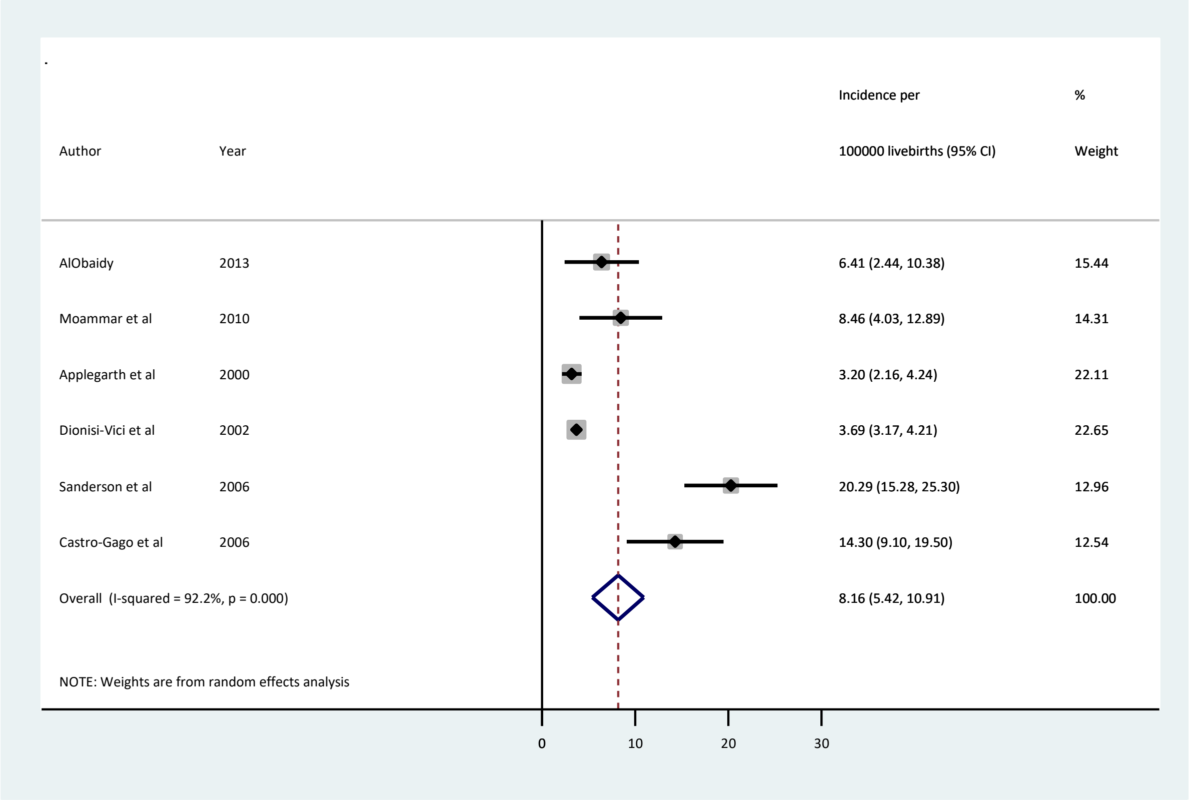

A 3.16. Meta-estimate of incidence of all peroxisomal disorders

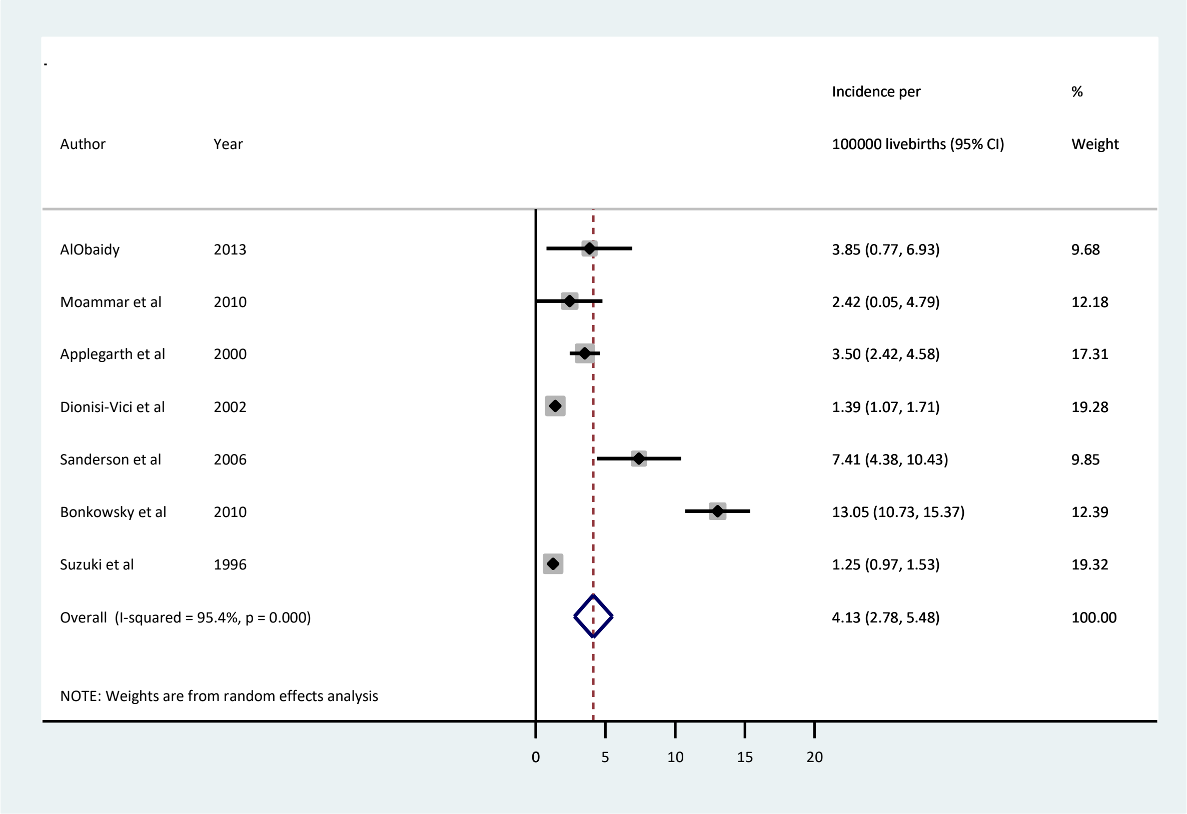

Appendix 4: characteristics of studies included for analysis of specific IEM categories

A 4.1. Studies reporting estimates for amino acid disorders

Author	Year	Location	Setting	Period of study	Total live births	Cases in cohort	Birth prevalence /100000 LB	Deaths in birth cohort
Al Bu Ali et al[1]	2011	Saudi Arabia, Al Ahsa	Urban-Rural	Apr 2006 - Apr 2009	38001	1	2.63	9
Huang et al[2]	2012	China, Zhejiang Province	Urban-Rural	Jan 2008 - Jan 2011		11		
Al Riyami et al[3]	2012	Oman, Al Khoudh	Urban-Rural	May 1998 - Jul 2008	241757	8	3.31	
Feuchtbaum et al[4]	2012	USA, California	Urban	Jul 2005 - Jul 2010	2282138	66	2.9	
Golbahar et al[5]	2013	Bahrain	Urban-Rural	Jan 2008 - Dec 2011	66565	3	4.51	
Moammar et al[6]	2010	Saudi Arabia, Dhahran Eastern Province	Urban-Rural	Jan 1983 - Jan 2008	165530	12	7.25	
Applegarth et al[7]	2000	Canada, British Columbia	Urban	Jan 1969- Jan 1996	1142912	86	7.10	
Sanderson et al[8]	2006	UK, West Midlands	Urban	Jan 1999 - Dec 2003	310510	25	8.05153	
Nagaraja et al[9]	2010	India, Southern states	Urban-Rural	Jan 2007 - Dec 2009		19		
Shigematsu et al[10]	2002	Japan, Fukui	Urban-Rural	Apr 1997 - Jul 2001	102200	1	0.978474	137
Yoon et al[11]	2005	South Korea, Seoul	Urban	Apr 2001 - Mar 2004	79179	4	5.05	
Abadie et al[12]	2001	France, National	Urban-Rural	1980-1997	21500000	1426	5.84	5
Boneh et al[13]	2006	Australia, Victoria	Urban	Nov 2001- Oct 2004	190835	17	8.9	
Jiang et al[14]	2003	China, Guangdong	Urban-Rural	2001-2002	461805	14	1.3	
Lugovska et al[15]	1999	Latvia	Urban-Rural	1980-1998	38684	51	11.49	
Oddason et al[16]	2011	Iceland, National	Urban-Rural	1972-2008	226891	27	11.9	
Chen et al[17]	1989	China, Shanghai	Urban	Oct 1981- Dec 1988	358767	21	5.88	3
Schuler et al[18]	1996	Hungary, Budapest	Urban	Jan 1975 - Dec 1994	1500000	160	11.11	
Zhao et al[19]	2005	China, Zhejiang province	Urban-Rural	Jan 2002- Jan 2003	726998	32	4.40	
Hardelid et al[20]	2008	England, South East	Urban	Jan 1994-Dec 2004	1576419	167	10.59	
Sander et al[21]	2001	Germany	Urban	Jul 1989 - Dec 1999	283408	28	10	
Zhan et al[22]	2009	China, Hangzhou	Urban-Rural	Jan 1985 - Dec 2007	19000000	1638	8.64	2
Couce et al[23]	2011	Spain, Galicia	Urban	Jul 2000 - Jul 2010	210165	17	8.09	
Frazier et al[24]	2006	USA, North Carolina	Urban	Jul 1997- Jul 2005	944078	49	5.19	

Kasper et al[25]	2010	Austria, National	Urban-Rural	Apr 2002 - Dec 2009	622489	50	8.03	1
Niu et al[26]	2010	Taiwan	Urban-Rural	Mar 2000 - Jun 2009	1495132	25	1.67	
Schulze et al[27]	2003	Germany	Urban-Rural	Apr 1998 - Sep 2001	250000	24	9.60	
Selim et al[28]	2014	Egypt	Urban-Rural	Jun 2008 - Jun 2013	1100000	100	9.09	
Tan et al[29]	2006	Singapore	Urban	Jan 1992 -Jan 2005	40800	6	14.71	
Vilarinho et al[30]	2010	Portugal, national	Urban-Rural	Jan 2005 - Jan 2009	316243	26	8.22	

A 4.2. Studies reporting estimates for organic acid disorders

Author	Year	Location	Setting	Period of study	Total live births	Cases in cohort	Birth prevalence /100000 LB	Deaths in birth cohort
AlObaidy[31]	2013	Libya, Tripoli	Urban	Jan 2001 - Dec 2012	156006	10	6.41	1
Al Bu Ali et al[1]	2011	Saudi Arabia, Al Ahsa	Urban-Rural	Apr 2006 - Apr 2009	38001	10	26.32	
Huang et al[2]	2012	China, Zhejiang Province	Urban-Rural	Jan 2008 - Jan 2011		26		5
Al Riyami et al[3]	2012	Oman, Al Khoudh	Urban-Rural	May 1998 - Jul 2008	241757	35	14.48	
Feuchtbaum et al[4]	2012	USA, California	Urban	Jul 2005 - Jul 2010	2282138	221	9.7	
Golbahar et al[5]	2013	Bahrain	Urban-Rural	Jan 2008 - Dec 2011	66565	9	13.52	
Kamate et al[32]	2010	India, Karnataka	Urban-Rural	Aug 2007 - Sept 2008		4		2
Moammar et al[6]	2010	Saudi Arabia, Dhahran Eastern Province	Urban-Rural	Jan 1983 - Jan 2008	165530	48	29.00	
Applegarth et al[7]	2000	Canada, British Columbia	Urban	Jan 1969- Jan 1996	1142912	36	4.60	
Dionisi-Vici et al[33]	2002	Italy, national survey	Urban-Rural	Jan 1985 - Dec 1997	5336730	249	4.67	58
Sanderson et al[8]	2006	UK, West Midlands	Urban	Jan 1999 - Dec 2003	310510	39	12.55966	
Nagaraja et al[9]	2010	India, Southern states	Urban-Rural	Jan 2007 - Dec 2009		47		
Han et al[34]	2007	China, Shanghai	Urban	Jan 2002 - Oct 2006		107		
Huang et al[35]	2011	China, Zhejiang province	Urban-Rural	Jan 2008 - Jan 2011	129415	6	4.64	
Yoon et al[11]	2005	South Korea, Seoul	Urban	Apr 2001 - Mar 2004	79179	10	12.63	
Hadj-Taieb et al[36]	2012	Tunisia, Tunis	Urban	Jan 1987- Dec 2009	946404	158	16.69	
Klose et al[37]	2002	Germany	Urban-Rural	Jan 1999- Dec 2000	844575	30	3.55	3
Couce et al[23]	2011	Spain, Galicia	Urban	Jul 2000 - Jul 2010	210165	24	11.42	3
Frazier et al[24]	2006	USA, North Carolina	Urban	Jul 1997- Jul 2005	944078	58	6.14	3
Kasper et al[25]	2010	Austria, National	Urban-Rural	Apr 2002 - Dec 2009	622489	46	7.39	
Niu et al[26]	2010	Taiwan	Urban-Rural	Mar 2000 - Jun 2009	1495132	51	3.41	
Schulze et al[27]	2003	Germany	Urban-Rural	Apr 1998 - Sep 2001	250000	17	6.80	
Selim et al[28]	2014	Egypt	Urban-Rural	Jun 2008 - Jun 2013	1100000	69	6.27	
Tan et al[29]	2006	Singapore	Urban	Jan 1992 -Jan 2005	40800	41	100.49	
Vilarinho et al[30]	2010	Portugal, national	Urban-Rural	Jan 2005 - Jan 2009	316243	24	7.59	
Wilcken et al[38]	2003	Australia, New South Wales / Sydney	Urban	April 1998 - Mar 2002	362000	53	14.64	

Wilcken et al[39]	2009	Australia, national	Urban-Rural	Jan 1994 - Jan2002	1551200	40	2.58	

A 4.3. Studies reporting estimates for fatty acid disorders

Author	Year	Location	Setting	Period of study	Total live births	Cases in cohort	Birth prevalence /100000 LB	Deaths in birth cohort
AlObaidy[31]	2013	Libya, Tripoli	Urban	Jan 2001 - Dec 2012	156006	4	2.56	3
Al Bu Ali et al[1]	2011	Saudi Arabia, Al Ahsa	Urban-Rural	Apr 2006 - Apr 2009	38001	31	81.58	
Huang et al[2]	2012	China, Zhejiang Province	Urban-Rural	Jan 2008 - Jan 2011		9		
Al Riyami et al[3]	2012	Oman, Al Khoudh	Urban-Rural	May 1998 - Jul 2008	241757	22	9.10	
Feuchtbaum et al[4]	2012	USA, California	Urban	Jul 2005 - Jul 2010	2282138	260	11.4	
Golbahar et al[5]	2013	Bahrain	Urban-Rural	Jan 2008 - Dec 2011	66565	5	7.51	
Kamate et al[32]	2010	India, Karnataka	Urban-Rural	Aug 2007 - Sept 2008		6		2
Moammar et al[6]	2010	Saudi Arabia, Dhahran Eastern Province	Urban-Rural	Jan 1983 - Jan 2008	165530	18	10.87	
Dionisi-Vici et al[33]	2002	Italy, national survey	Urban-Rural	Jan 1985 - Dec 1997	5336730	58	1.09	
Sanderson et al[8]	2006	UK, West Midlands	Urban	Jan 1999 - Dec 2003	310510	24	7.72917	
Nagaraja et al[9]	2010	India, Southern states	Urban-Rural	Jan 2007 - Dec 2009		5		
Han et al[34]	2007	China, Shanghai	Urban	Jan 2002 - Oct 2006		13		
Huang et al[35]	2011	China, Zhejiang province	Urban-Rural	Jan 2008 - Jan 2011	129415	4	3.09	
Shigematsu et al[10]	2002	Japan, Fukui	Urban-Rural	Apr 1997 - Jul 2001	102200	2	1.956947	
Tu et al [40]	2012	China, Beijing	Urban	Jan - Dec 2009	8211	1	12.18	
Yoon et al[11]	2005	South Korea, Seoul	Urban	Apr 2001 - Mar 2004	79179	5	6.31	
Klose et al[37]	2002	Germany	Urban-Rural	Jan 1999- Dec 2000	844575	27	3.20	5
Couce et al[23]	2011	Spain, Galicia	Urban	Jul 2000 - Jul 2010	210165	18	8.56	1
Frazier et al[24]	2006	USA, North Carolina	Urban	Jul 1997- Jul 2005	944078	99	10.49	2
Kasper et al[25]	2010	Austria, National	Urban-Rural	Apr 2002 - Dec 2009	622489	47	7.55	
Niu et al[26]	2010	Taiwan	Urban-Rural	Mar 2000 - Jun 2009	1495132	12	0.80	
Schulze et al[27]	2003	Germany	Urban-Rural	Apr 1998 - Sep 2001	250000	24	9.60	2
Selim et al[28]	2014	Egypt	Urban-Rural	Jun 2008 - Jun 2013	1100000	7	0.64	
Vilarinho et al[30]	2010	Portugal, national	Urban-Rural	Jan 2005 - Jan 2009	316243	50	15.81	
Wilcken et al[38]	2003	Australia, New South Wales / Sydney	Urban	April 1998 - Mar 2002	362000	67	18.51	
Wilcken et al[39]	2009	Australia, national	Urban-Rural	Jan 1994 - Jan2002	1551200	42	2.71	6

A 4.4. Studies reporting estimates for lysosomal disorders

Author	Year	Location	Setting	Period of study	Total live births	Cases in cohort	Birth prevalence /100000 LB	Deaths in birth cohort
AlObaidy[31]	2013	Libya, Tripoli	Urban	Jan 2001 - Dec 2012	156006	15	9.62	8
Goel et al[41]	2010	Australia, Victoria	Urban	Jan 1997 - Jan 2007				21
Moammar et al[6]	2010	Saudi Arabia, Dhahran Eastern Province	Urban-Rural	Jan 1983 - Jan 2008	165530	74	44.70	
Applegarth et al[7]	2000	Canada, British Columbia	Urban	Jan 1969- Jan 1996	1142912	70	7.60	
Dionisi-Vici et al[33]	2002	Italy, national survey	Urban-Rural	Jan 1985 - Dec 1997	5336730	645	12.08	
Sanderson et al[8]	2006	UK, West Midlands	Urban	Jan 1999 - Dec 2003	310510	60	19.32367	
Meikle et al[42]	1999	Australia, national	Urban	Jan 1980 - Dec 1996	4222323	470	11.13	
Malm et al[43]	2008	Norway	Urban - Rural	1975-2004	1461039	45	3.08	
Malm et al[43]	2008	Denmark	Urban - Rural	1975-2004	1864407	33	1.77	
Mechtler et al[44]	2012	Austria	Urban - Rural	Jan-Dec 2010	34736	15	43.18	
Poupetova et al[45]	2010	Czech Rep	Urban	1975-2008	4326531	530	12.25	
Tan et al[29]	2006	Singapore	Urban	Jan 1992 -Jan 2005	40800	15	36.76	

A 4.4. Studies reporting estimates for carbohydrate metabolism disorders

Author	Year	Location	Setting	Period of study	Total live births	Cases in cohort	Birth prevalence /100000 LB	Deaths in birth cohort
AlObaidy[31]	2013	Libya, Tripoli	Urban	Jan 2001 - Dec 2012	156006	16	14.44	4
Al Bu Ali et al[1]	2011	Saudi Arabia, Al Ahsa	Urban-Rural	Apr 2006 - Apr 2009	38001	2	5.26	
Moammar et al[6]	2010	Saudi Arabia, Dhahran Eastern Province	Urban-Rural	Jan 1983 - Jan 2008	165530	16	9.67	
Applegarth et al[7]	2000	Canada, British Columbia	Urban	Jan 1969- Jan 1996	1142912	40	3.80	
Dionisi-Vici et al[33]	2002	Italy, national survey	Urban-Rural	Jan 1985 - Dec 1997	5336730	273	5.12	2
Sanderson et al[8]	2006	UK, West Midlands	Urban	Jan 1999 - Dec 2003	310510	19	6.118828	
Tan et al[29]	2006	Singapore	Urban	Jan 1992 -Jan 2005	40800	6	14.71	

A 4.5. Studies reporting estimates for urea cycle disorders

Author	Year	Location	Setting	Period of study	Total live births	Cases in cohort	incidence/100000 livebirths	Deaths in birth cohort
AlObaidy[31]	2013	Libya, Tripoli	Urban	Jan 2001 - Dec 2012	156006	3	1.92	
Al Bu Ali et al[1]	2011	Saudi Arabia, Al Ahsa	Urban-Rural	Apr 2006 - Apr 2009	38001	1	2.63	
Moammar et al[6]	2010	Saudi Arabia, Dhahran Eastern Province	Urban-Rural	Jan 1983 - Jan 2008	165530	12	7.25	
Applegarth et al[7]	2000	Canada, British Columbia	Urban	Jan 1969- Jan 1996	1142912	18	1.90	
Dionisi-Vici et al[33]	2002	Italy, national survey	Urban-Rural	Jan 1985 - Dec 1997	5336730	129	2.41	
Sanderson et al[8]	2006	UK, West Midlands	Urban	Jan 1999 - Dec 2003	310510	14	4.51	
Ibarra- Gonzalez et al[46]	2014	Mexico, Mexico city	Urban	Jan 2007 - Dec 2012		2		2
Couce et al[23]	2011	Spain, Galicia	Urban	Jul 2000 - Jul 2010	210165	3	1.43	
Tan et al[29]	2006	Singapore	Urban	Jan 1992 -Jan 2005	40800	14	34.31	
Vilarinho et al[30]	2010	Portugal, national	Urban-Rural	Jan 2005 - Jan 2009	316243	4	1.26	
Wilcken et al[38]	2003	Australia, New South Wales / Sydney	Urban	April 1998 - Mar 2002	362000	44	12.15	
Wilcken et al[39]	2009	Australia, national	Urban-Rural	Jan 1994 - Jan2002	1551200	23	1.48	

A 4.6. Studies reporting estimates for mitochondrial disorders

Author	Year	Location	Setting	Period of study	Total live births	Cases in cohort	Birth prevalence /100000 LB	Deaths in birth cohort
AlObaidy[31]	2013	Libya, Tripoli	Urban	Jan 2001 - Dec 2012	156006	10	6.41	6
Goel et al[41]	2010	Australia, Victoria	Urban	Jan 1997 - Jan 2007				29
Moammar et al[6]	2010	Saudi Arabia, Dhahran Eastern Province	Urban-Rural	Jan 1983 - Jan 2008	165530	14	8.46	
Applegarth et al[7]	2000	Canada, British Columbia	Urban	Jan 1969- Jan 1996	1142912	13	3.20	
Dionisi-Vici et al[33]	2002	Italy, national survey	Urban-Rural	Jan 1985 - Dec 1997	5336730	197	3.69	76
Sanderson et al[8]	2006	UK, West Midlands	Urban	Jan 1999 - Dec 2003	310510	63	20.28809	
Castro-Gago et al[47]	2006	Spain, North West	Urban-Rural	Jan 1990- Dec 1999	203054	29	14.3	16

A 4.7. Studies reporting estimates for peroxisomal disorders

Author	Year	Location	Setting	Period of study	Total live births	Cases in cohort	Birth prevalence /100000 LB	Deaths in birth cohort
AlObaidy[31]	2013	Libya, Tripoli	Urban	Jan 2001 - Dec 2012	156006	6	3.85	3
Goel et al[41]	2010	Australia, Victoria	Urban	Jan 1997 - Jan 2007				6
Moammar et al[6]	2010	Saudi Arabia, Dhahran Eastern Province	Urban-Rural	Jan 1983 - Jan 2008	165530	4	2.42	
Applegarth et al[7]	2000	Canada, British Columbia	Urban	Jan 1969- Jan 1996	1142912	20	3.50	
Dionisi-Vici et al[33]	2002	Italy, national survey	Urban-Rural	Jan 1985 - Dec 1997	5336730	74	1.39	
Sanderson et al[8]	2006	UK, West Midlands	Urban	Jan 1999 - Dec 2003	310510	23	7.407407	
Bonkowsky et al[48]	2010	USA, Utah	Urban	Jan 1999 - Dec 2007	934886	122	13.05	42
Suzuki et al[49]	1996	Japan	Urban-Rural		6000000	75	1.25	
Tan et al[29]	2006	Singapore	Urban	Jan 1992 -Jan 2005		3	7.35	

References (for all studies selected).

[1] Al Bu Ali WH, Balaha MH, Al Moghannum MS, Hashim I. Risk factors and birth prevalence of birth defects and inborn errors of metabolism in Al Ahsa, Saudi Arabia. Pan Afr Med J 2011;8:14.

[2] Huang X, Yang L, Tong F, Yang R, Zhao Z. Screening for inborn errors of metabolism in high-risk children: a 3-year pilot study in Zhejiang Province, China. BMC Pediatr 2012;12:18.

[3] Al-Riyami S, Maney M Al, Joshi SN, Bayoumi R. Detection of Inborn Errors of Metabolism using Tandem Mass Spectrometry among High-risk Omani Patients. Oman Med J 2012;47:482–5.

[4] Feuchtbaum L, Carter J, Dowray S, Currier RJ, Lorey F. Birth prevalence of disorders detectable through newborn screening by race/ethnicity. Genet Med 2012;14:937–45.

[5] Golbahar J, Al-Jishi EA, Altayab DD, Carreon E, Bakhiet M, Alkhayyat H. Selective newborn screening of inborn errors of amino acids, organic acids and fatty acids metabolism in the Kingdom of Bahrain. Mol Genet Metab 2013;110:98–101.

[6] Moammar H, Cheriyan G, Mathew R, Al-Sannaa N. Incidence and patterns of inborn errors of metabolism in the Eastern Province of Saudi Arabia, 1983-2008. Ann Saudi Med 2010;30:271. doi:10.4103/0256-4947.65254.

[7] Applegarth DA, Toone JR, Lowry RB. Incidence of inborn errors of metabolism in British Columbia, 1969-1996. Pediatrics 2000;105:e10.

[8] Sanderson S, Green A, Preece MA, Burton H. The incidence of inherited metabolic disorders in the West Midlands, UK. Arch Dis Child 2006;91:896–9.

[9] Nagaraja D, Mamatha SN, De T, Christopher R. Screening for inborn errors of metabolism using automated electrospray tandem mass spectrometry: Study in high-risk Indian population. Clin Biochem 2010;43:581–8.

[10] Shigematsu Y, Hirano S, Hata I, Tanaka Y, Sudo M, Sakura N, et al. Newborn mass screening and selective screening using electrospray tandem mass spectrometry in Japan. J Chromatogr B Analyt Technol Biomed Life Sci 2002;776:39–48.

[11] Yoon H-R, Lee KR, Kang S, Lee DH, Yoo H-W, Min W-K, et al. Screening of newborns and high-risk group of children for inborn metabolic disorders using tandem mass spectrometry in South Korea: a three-year report. Clin Chim Acta 2005;354:167–80. doi:10.1016/j.cccn.2004.11.032.

[12] Abadie V, Berthelot J, Feillet F, Maurin N, Mercier A, de Baulny HO, et al. Neonatal screening and long-term follow-up of phenylketonuria: the French database. Early Hum Dev 2001;65:149–58.

[13] Boneh A, Francis DE, Humphrey M, Upton HJ, Peters HL. Three-year audit of the hyperphenylalaninaemia/phenylketonuria spectrum in Victoria. J Paediatr Child Heal 2006;42:496–8.

[14] Jiang J, Ma X, Huang X, Pei X, Liu H, Tan Z, et al. A survey for the incidence of phenylketonuria in Guangdong, China. Southeast Asian J Trop Med Public Heal 2003;34 Suppl 3:185.

[15] Lugovska R, Vevere P, Andrusaite R, Kornejeva A. Newborn screening for PKU and congenital hypothyroidism in Latvia. Southeast Asian J

Trop Med Public Heal 1999;30 Suppl 2:52–3.
[16] Oddason KE, Eiriksdottir L, Franzson L, Dagbjartsson A. [Phenylketonuria (PKU) in Iceland]. Laeknabladid 2011;97:349–52.
[17] Chen RG, Pan XS, Qian DL, Guo H. Twenty-one cases of phenylketonuria out of 358,767 newborns in Shanghai, China. J Inherit Metab Dis 1989;12:485.
[18] Schuler A, Somogyi C, Toros I, Pataki L, Mete M, Kiss E, et al. A longitudinal study of phenylketonuria based on the data of the Budapest Screening Center. Eur J Pediatr 1996;155 Suppl 1:S50-2.
[19] Zhao ZY, Qu YP, Qu LQ, Yu XL. [Screening for phenylketonuria in 726,998 neonates in Zhejiang Province]. Zhejiang da Xue Xue Bao Yi Xue Ban/Journal Zhejiang Univ Med Sci 2005;34:185–7.
[20] Hardelid P, Cortina-Borja M, Munro A, Jones H, Cleary M, Champion MP, et al. The birth prevalence of PKU in populations of European, South Asian and sub-Saharan African ancestry living in South East England. Ann Hum Genet 2008;72:65–71. doi:http://dx.doi.org/10.1111/j.1469-1809.2007.00389.x.
[21] Sander S, Janzen N, Janetzky B, Scholl S, Steuerwald U, Schafer J, et al. Neonatal screening for medium chain acyl-CoA deficiency: high incidence in Lower Saxony (northern Germany). Eur J Pediatr 2001;160:318–9.
[22] Zhan JY, Qin YF, Zhao ZY. Neonatal screening for congenital hypothyroidism and phenylketonuria in China. World J Pediatr 2009;5:136–9. doi:http://dx.doi.org/10.1007/s12519-009-0027-0.
[23] Couce ML, Bana A, Boveda MD, Perez-Munuzuri A, Fernandez-Lorenzo JR, Fraga JM. Inborn errors of metabolism in a neonatology unit: impact and long-term results. Pediatr Int 2011;53:13–7. doi:http://dx.doi.org/10.1111/j.1442-200X.2010.03177.x.
[24] Frazier DM, Millington DS, McCandless SE, Koeberl DD, Weavil SD, Chaing SH, et al. The tandem mass spectrometry newborn screening experience in North Carolina: 1997-2005. J Inherit Metab Dis 2006;29:76–85.
[25] Kasper DC, Ratschmann R, Metz TF, Mechtler TP, Möslinger D, Konstantopoulou V, et al. The National Austrian Newborn Screening Program – Eight years experience with mass spectrometry. Past, present, and future goals. Wien Klin Wochenschr 2010;122:607–13. doi:10.1007/s00508-010-1457-3.
[26] Niu DM, Chien YH, Chiang CC, Ho HC, Hwu WL, Kao SM, et al. Nationwide survey of extended newborn screening by tandem mass spectrometry in Taiwan. J Inherit Metab Dis 2010;33:S295-305. doi:http://dx.doi.org/10.1007/s10545-010-9129-z.
[27] Schulze A, Lindner M, Kohlmüller D, Olgemöller K, Mayatepek E, Hoffmann GF. Expanded newborn screening for inborn errors of metabolism by electrospray ionization-tandem mass spectrometry: results, outcome, and implications. vol. 111. 2003. doi:10.1007/bf01799385.
[28] Selim L a., Hassan SAH, Salem F, Orabi A, Hassan F a., El-Mougy F, et al. Selective screening for inborn errors of metabolism by tandem mass spectrometry in Egyptian children: A 5year report. Clin Biochem 2014;47:823–8. doi:10.1016/j.clinbiochem.2014.04.002.
[29] Tan IK, Gajra B, Lim MS. Study of inherited metabolic disorders in Singapore - 13 years experience. Ann Acad Med Singapore

2006;35:804–13.
[30] Vilarinho L, Rocha H, Sousa C, Marcao A, Fonseca H, Bogas M, et al. Four years of expanded newborn screening in Portugal with tandem mass spectrometry. J Inherit Metab Dis 2010;33 Suppl 3:S133-8. doi:http://dx.doi.org/10.1007/s10545-010-9048-z.
[31] AlObaidy H. Patterns of inborn errors of metabolism: A 12 year single-center hospital-based study in Libya. Qatar Med J 2013;18:1–9.
[32] Kamate M, Chetal V, Kulgod V, Patil V, Christopher R. Profile of inborn errors of metabolism in a tertiary care centre PICU. Indian J Pediatr 2010;77:57–60. doi:10.1007/s12098-010-0008-2.
[33] Dionisi-Vici C, Rizzo C, Burlina AB, Caruso U, Sabetta G, Uziel G, et al. Inborn errors of metabolism in the Italian pediatric population: a national retrospective survey. J Pediatr 2002;140:321–7.
[34] Han LS, Ye J, Qiu WJ, Gao XL, Wang Y, Gu XF. Selective screening for inborn errors of metabolism on clinical patients using tandem mass spectrometry in China: A four-year report. J Inherit Metab Dis 2007;30:507–14.
[35] Huang X, Yang J, Tong F, Yang R, Mao H, Zhou X, et al. [Screening for neonatal inborn errors of metabolism by electrospray ionization-tandem mass spectrometry and follow-up]. Zhonghua Er Ke Za Zhi = Chinese J Pediatr 2011;49:765–70.
[36] Hadj-Taieb S, Nasrallah F, Hammami MB, Elasmi M, Sanhaji H, Moncef F, et al. Aminoacidopathies and organic acidurias in Tunisia: a retrospective survey over 23 years. Tunisie Medicale 2012;90:258–61.
[37] Klose DA, Kolker S, Heinrich B, Prietsch V, Mayatepek E, von Kries R, et al. Incidence and short-term outcome of children with symptomatic presentation of organic acid and fatty acid oxidation disorders in Germany. Pediatrics 2002;110:1204–11.
[38] Wilcken B, Wiley V, Hammond J, Carpenter K. Screening newborns for inborn errors of metabolism by tandem mass spectrometry. N Engl J Med 2003;348:2304–12.
[39] Wilcken B, Haas M, Joy P, Wiley V, Bowling F, Carpenter K, et al. Expanded newborn screening: outcome in screened and unscreened patients at age 6 years. Pediatrics 2009;124:e241-8. doi:http://dx.doi.org/10.1542/peds.2008-0586.
[40] Tu W, He J, Dai F, Wang X, Li Y. Impact of Inborn Errors of Metabolism on Admission in a Neonatal Intensive Care Unit—A Prospective Cohort Study. Indian J Pediatr 2012;79:494–500. doi:10.1007/s12098-011-0464-3.
[41] Goel H, Lusher A, Boneh A. Pediatric Mortality Due to Inborn Errors of Metabolism in Victoria, Australia: A Population-Based Study. JAMA 2010;304:1070. doi:10.1001/jama.2010.1259.
[42] Meikle PJ, Ranieri E, Ravenscroft EM, Hua CT, Brooks DA, Hopwood JJ. Newborn screening for lysosomal storage disorders. Southeast Asian J Trop Med Public Heal 1999;30 Suppl 2:104–10.
[43] Malm G, Lund AM, Mansson JE, Heiberg A. Mucopolysaccharidoses in the Scandinavian countries: incidence and prevalence.[Erratum appears in Acta Paediatr. 2008 Dec;97(12):1773]. Acta Paediatr 2008;97:1577–81. doi:http://dx.doi.org/10.1111/j.1651-2227.2008.00965.x.
[44] Mechtler TP, Stary S, Metz TF, De Jesus VR, Greber-Platzer S, Pollak A, et al. Neonatal screening for lysosomal storage disorders:

feasibility and incidence from a nationwide study in Austria. Lancet 2012;379:335–41. doi:http://dx.doi.org/10.1016/S0140-6736(11)61266-X.
[45] Poupetova H, Ledvinova J, Berna L, Dvorakova L, Kozich V, Elleder M. The birth prevalence of lysosomal storage disorders in the Czech Republic: comparison with data in different populations. J Inherit Metab Dis 2010;33:387–96. doi:http://dx.doi.org/10.1007/s10545-010-9093-7.
[46] Ibarra-González I, Fernández-Lainez C, Reyes-González D, Belmont-Martínez L, Guillén-López S, Monroy-Santoyo S, et al. Inborn Errors of Intermediary Metabolism in Critically Ill Mexican Newborns. J Inborn Errors Metab Screen 2014;2:232640981452964. doi:10.1177/2326409814529649.
[47] Castro-Gago M, Blanco-Barca MO, Campos-Gonzalez Y, Arenas-Barbero J, Pintos-Martinez E, Eiris-Punal J. Epidemiology of pediatric mitochondrial respiratory chain disorders in northwest Spain. Pediatr Neurol 2006;34:204–11.
[48] Bonkowsky JL, Nelson C, Kingston JL, Filloux FM, Mundorff MB, Srivastava R. The burden of inherited leukodystrophies in children. Neurology 2010;75:718–25. doi:http://dx.doi.org/10.1212/WNL.0b013e3181eee46b.
[49] Suzuki Y, Shimozawa N, Yajima S, Inoue K, Orii T, Kondo N. Incidence of peroxisomal disorders in Japan. Jpn J Hum Genet 1996;41:167–75.
